# Incloodle-Classroom: Technology for Inclusive Joint Media Engagement in a Neurodiverse Kindergarten Classroom

**DOI:** 10.1145/3674506

**Published:** 2024

**Authors:** KILEY SOBEL, MAITRAYE DAS, SARA BEHBAKHT, JULIE A. KIENTZ

**Affiliations:** University of Washington, Seattle, WA, USA; Northeastern University, Boston, MA, USA; IDEO, Seattle, WA, USA; University of Washington, Seattle, WA, USA

**Keywords:** Inclusive play, inclusive classroom, joint media engagement, neurodiversity, kindergarten, children, child-computer interaction

## Abstract

Enabling opportunities for young children with disabilities to co-engage in learning activities alongside their non-disabled peers is essential for promoting equity in early childhood education. We investigate how collaborative technology can be designed to support young neurodivergent and neurotypical children in playing together. By integrating theories and methods from design, HCI, and the learning sciences, we iteratively designed, developed, and evaluated a novel tablet application called Incloodle-Classroom (Incloodle in short), that takes into account the needs of neurodiverse groups of children and the adults who support them during play. We deployed Incloodle in a kindergarten classroom of 15 neurodivergent and 16 neurotypical children over a 10-week period. Using interaction analysis, we present rich empirical understandings of how children interacted with each other, with adults, and with Incloodle. In doing so, we contribute new theoretical underpinnings to collaborative and accessible technology design, extending joint media engagement to encompass inclusivity and equity.

## 1 Introduction

In the **human–computer interaction (HCI)** research and design communities, there has been a critical movement toward increasing the accessibility of technologies and continually addressing ways to ensure that people with disabilities can fully access, participate in, and engage with technologies. This becomes a distinctive, complex question when we also consider cooperative engagement with technologies—when two or more people use technologies together. Cooperative engagement with technology, also known as **joint media engagement (JME)** [38], is particularly important when designing for children [22]. This is because by jointly viewing, playing, searching, reading, contributing to, and creating with digital media, children have the context and resources to create meaningful connections among representations, interests, and experiences [81]. When children jointly engage with digital media with other children or adults, regardless of if that digital media is traditionally “educational” or not, they are afforded opportunities to learn [81]. This learning is not only in regard to the media being consumed but also involves social, cognitive, and emotional benefits [81].

While researchers have studied JME in families with neurotypical children and adults [e.g., 24, 33, 76], leading to technology design principles for children’s productive JME [81], it is not yet known how these principles might change or be supplemented when also taking into account disability and neurodivergence. Drawing on inclusive education and accessibility research informed by the disability studies literature [e.g., 41, 52, 75], we explore how to make cooperative interactive technology experiences more inclusive for children with and without disabilities. We ask how technologies might be designed so that all children can meaningfully participate and engage with them together and with adults, despite differences in their physical, cognitive, behavioral, social, or emotional abilities and needs. We refer to this as designing technologies for *inclusive JME*. Beyond the typical benefits of JME, with inclusive JME, children have additional social and emotional learning opportunities associated with inclusion, such as developing empathy [61], learning to “be with” [9] each other, cultivating a positive mindset about disabilities, and feeling accepted and part of a community [66]. In this article, we describe research that builds upon our prior formative work in inclusive play [78] to design, develop, and evaluate a technology for promoting Inclusive JME among kindergarten students in an inclusive classroom setting.

Our research aims to answer the broader research question: *how do interactions between neurodiverse*^1^ *groups of children, adults, and technology shape inclusive play in a formal learning environment?* As part of this larger question, we address two interrelated sub-questions:

— RQ1: What types of interactions among neurodiverse children (that were or were not necessarily designed for) emerge over time during collaborative play with a tablet application and adult support?

— RQ2: How does this joint socio-material context engender or restrict equitable engagement and participation (i.e., inclusion) during moments of interaction with the technology over time? Who and what does the “including” (or “excluding”) in this context?

To address these questions, we completely redesigned an application we developed earlier for promoting inclusive play [79] and deployed it as an intervention for an inclusive classroom. Our system, called *Incloodle-Classroom* or *Incloodle* in short,^2^ is a tablet-based picture-taking application that uses diverse social characters who use stories to encourage children to ask each other questions, engage in social-emotional learning activities, and take pictures together. There is a built-in reward mechanism where children can collaborate to decorate their pictures and print them on stickers with an attached Bluetooth printer. We studied how 15 neurodivergent and 16 neurotypical kindergarteners co-played with the application in a classroom setting over a 10-week deployment period. Using methods of interaction analysis [35, 48, 64] on the video data collected over this period, we developed six interconnected themes that describe (1) connections and reflections on children’s characteristics and experiences, (2) making sense of and (not) following the application prompts, (3) negotiation of physical and virtual spaces, (4) adult scaffolding as integral to the Incloodle system, (5) significance of the process and product of printing pictures, and (6) pictures as “proof” of inclusion vs. the “work” of inclusion behind the picture.

Overall, our research makes three key contributions. Our first contribution is *Incloodle* as a fully functional artifact [86] that embeds values of inclusion and equity into its design and works as an intervention for inclusive play and social-emotional learning among young children. Second, through a micro-analysis of child-to-child, adult-to-child (and vice-versa), and child-to-technology (and vice-versa) interactions over an extended period of time, we unpack the ways in which Incloodle was positioned as a mediational tool, structuring artifact, and semiotic resource in regard to inclusive play in a classroom setting. Our analysis offers evidence of children’s joint learning of how to be inclusive spatially, communicatively, and in interaction/engagement with, around, and through technology, often with adult support. Finally, we discuss ways to rethink collaborative and accessible technology design for neurodiverse groups of children and JME, expanding these notions to consider inclusion as continual reflection on equity and meaningful co-participation.

## 2 Background and Related Work

Our research is informed by the theoretical framings of JME, successful inclusion, and endogenous approach to learning. In addition, we draw on related HCI and Computer Supported Cooperative Work research on designing technologies for inclusive play and classroom experience among children with and without disabilities.

### 2.1 Theoretical Framing

#### 2.1.1 Joint Media Engagement.

Joint Media Engagement or JME refers to the experiences of people using media together, which include viewing, playing, searching, reading, contributing, and creating with either digital or traditional media [22, 24, 38]. JME supports learning by providing context and resources for people to co-create meaningful connections among representations, interests, and experiences [33]. Through JME, participants can make sense and meaning together in a particular situation and for future situations [81]. While active co-play around technologies can be a form of JME, discussions and dialogic inquiry, co-viewing, and collaborations around technologies can also be meaningful forms of co-engagement. Takeuchi and Stevens [81] drew from past research and development to produce a set of design principles and conditions for meaningful, productive JME: active discussion, multiple planes of engagement, differentiation of roles, appeal to multiple generations, ability to gain experience and change, co-creation of shared experiences, and focus on content (not control). However, because there are developmental differences between adults and children, younger and older children as well as disabled and non-disabled populations, a one-size-fits-all perspective of design for JME can exclude users with atypical developmental abilities and interests. As a successful example, *Sesame Street* uses their characters to draw in young children and celebrity cameos to bring in adults [28]. Differentiation of roles refers to assigning roles to participants so that the tasks and content match up to the user’s experience and age. Distinct roles, particularly in cooperative and collaborative tasks, can motivate individuals to work together. Moving forward, Takeuchi and Stevens [81, p. 55] note an important area for future research is studying “the qualities of media design and deliberate use that encourage productive JME.” Following this call for research, we help build knowledge about what design can do to foster productive JME between children and adults with and without disabilities.

#### 2.1.2 Successful Inclusion.

When referring to the term “inclusive” in our research, we specifically align with inclusion from the field of education. Here, inclusion occurs when students with and without disabilities participate in the same setting [66]. Cross et al. [19] define “successful inclusion” as occurring when (1) children make progress on their individual goals; (2) children make progress in their personal development and in knowledge acquisition expected for all children; (3) children are welcomed and accepted as full members of the group; and (4) children’s parents or caregivers are satisfied with their children’s gains and how happy and comfortable their children are in the group. This definition can be adapted for specific children in specific settings or contexts. Along the same lines, Sandall et al. [74] offer a comprehensive framework of strategies to support positive peer interactions in the inclusive classroom. In this framework, four main “building blocks” can be utilized to facilitate friendships and social relationships: **child-focused instructional strategies (CFIS)**, embedded learning opportunities, curriculum modifications, and providing a high-quality early childhood environment. While CFIS concern the explicit teaching of concepts, the other three offer ways to modify and plan activities, materials, and the environment such that children are supported and encouraged to play and socialize with each other.

#### 2.1.3 Endogenous Approach to Learning.

Aligning with Stevens [80, pp. 95–96], we believe “children deserve the right to find, see, and make their own learning.” Hence, we analyze learning, participation, and interaction from an endogenous perspective as opposed to an exogenous one. An exogenous approach to learning, which most people are familiar with, examines learning “from without” or from perspectives “outside” the learner. In this approach, learners are often pre- and post-tested, according to specific dimensions or outcomes deemed important by authorities beyond the learners themselves. Stevens argue that an exogenous approach, as an “administrative science” concerned with capacities chosen by “normative institutional forces,” is “ill-suited to capture learning events that emerge in the practical concerns of everyday life” [80, p. 83].

Opposingly, an endogenous approach to learning examines learning “from within” or from the perspectives of the learners. In this view, learning is “co-constructed in and across events between people, and between people and things, in everyday life” [80, p. 83]. This way, learning is considered as a members’ phenomenon. As such, learning can be analyzed at the level of interactional events to establish that “an activity is for its participants a learning event” [80, p. 85]. When learning is approached as a members’ phenomenon, researchers can argue learning is happening by showing and interpreting how participants initiate, orient to, and sustain an interaction as a learning event, including the interactional resources they use to do so.

### 2.2 Interactive Technologies for Joint Media Engagement

HCI researchers have designed and evaluated various interactive technologies to support JME, particularly in regard to facilitating and encouraging communication, collaboration, and cooperative play between children (for an overview, see [44]). For example, interactive tabletop displays have altered how young children can engage together through augmented fantasy play [53] and have added scaffolding and interactivity to table-based activities like puzzles and path tracking [49]. Tangible user interfaces have also been a natural and successful way to bring children together [4], especially in the areas of collaborative programming [42] and storytelling [51, 54]. Additionally, large and small mobile devices have allowed children to collaboratively read and create stories [25], engage in spelling games in groups [46], and share and motivate each other to complete learning activities in a classroom [77]. Combined with augmented reality and cooperative problem solving, mobile games have also been able to facilitate physical coordination, physical support, and verbal instructions, clarifications, and communication between older-younger sibling pairs [6].

Families, particularly parents and children, have many different ways they can engage with each other around new technologies. For instance, Family Story Play is a system that successfully combines video communication, physical book reading, and popular media character Elmo to promote dialogic reading activities for very young children and their families across long distances [71]. Similarly, ShareTable is a “media space” for remote synchronous interaction, including video chat and a shared activity table, designed for children and their remote parents [87]. While new designs for JME are being created for remote interaction, there are still important engagements around technology that are local. In case study of Brooks et al. [14] with electric racer, a computer game specifically designed for two-player intergenerational play, parents and children needed roles within the game clarified, so that parents knew the game’s educational goals and how they were supposed to scaffold their children’s learning. Other researchers have found joint engagement around technology does not require even expertise between co-participants, which may be the case in families. In ethnographic study of Aarsand [3], uneven expertise between children and parents about video games actually fostered joint gaming. When children have more expertise and adults are novices, it allows children to take the lead and disrupt the typical balance of power between children and adults. Now, the introduction of location-based information, augmented reality, and virtual characters to interactive technologies have brought new JME opportunities to contexts in which boredom might ensue, such as long family car rides [40], or in which families may not have normally engaged together, like with Pokémon GO [76]. However, much of the prior research into JME has looked into the practices of neurotypical children and adult family members, meaning less is known about how technology can support inclusive JME between groups of children (and adults) when one or more group members experience disability or neurodivergence.

### 2.3 Designing for Inclusive Classroom Experience and Social Play

Within HCI, a burgeoning body of work has started investigating inclusive classroom experience for children with and without disabilities [60, 68]. As an example, researchers have explored the potential of robot technologies to promote inclusive education and collaborative learning among students with diverse visual abilities [57, 62, 63]. Metatla et al. engaged in a series of co-design studies with blind and sighted students in mainstream schools to build voice user interface applications [59] and collaborative multisensory storytelling tools [20]. These researchers identify “teaching assistance bubbles” as a central barrier to inclusive classroom environments, which can have detrimental effects on blind students’ engagement in group learning and social play [58]. Subsequently, they illustrate group dynamics that are conducive to collaborative learning, such as shared goal setting, tightly coupled division of labor, and interaction symmetry [57].

Closely related to our work are the studies that look into ways to facilitate inclusive play among neurodivergent children. Piper et al. [67] developed a cooperative tabletop game called SIDES to support autistic adolescents (age 11–14) practice and build confidence in their group work skills. Researchers have observed that tablet applications and games encourage autistic children to interact socially with each other (e.g., [13, 45, 89]). Others have focused on providing various techniques, materials, and ways to communicate to broaden participation of neurodivergent children in co-design activities. Drawing on the development process of a tangible playful prototype with “minimally-verbal” autistic children (aged 5–8) in an elementary school, Wilson et al. [85] put forth the “co-design beyond words” approach that highlights “moments of interaction” in which children can convey meaning through their actions and interactions such as joint attention, turn taking, and imitation. Through long-term participatory engagement with neurodiverse groups of children aged 6–8 years, Frauenberger et al. [30] designed a series of digital technologies that could scaffold and mediate social play activities among these children, such as pretend play and negotiating individual and group spaces. These researchers argue that designing with neurodiverse children requires careful mediation of spaces and structures to foster—rather than always resolve—constructive disagreements that naturally arise in a group with heterogeneous abilities, preferences, and conceptions of play [31]. Taking the lens of “double empathy,” Morris et al. [61] uncover perspectives of both autistic and neurotypical children in social play. Our research builds on these approaches that promote inclusion, but also focuses on a younger population (kindergarteners aged 4–7) and considers the greater education contexts in which children participate.

## 3 Incloodle-Classroom: Iterative System Design and Development

Our present article builds upon our formative ethnographic study [78] and the design and evaluation of *Incloodle 1.0* [79] to adapt and expand the ideas of the Incloodle system for a classroom setting and real world deployment.

### 3.1 Formative Ethnographic Study

Our investigation started with an ethnographic study on understanding opportunities for technology to promote inclusive play in classrooms [78]. Through a design ethnography of over eight months in two inclusive kindergarten classrooms along with surveys and interviews with teachers and parents of neurotypical and neurodivergent children, we identified a number of facilitators for inclusive play, including direct and embedded supports, transparency, adjustability, emphasis on children’s interests and strengths, and current technology use. We also identified significant barriers to inclusive play that technology must overcome, including the effort required to facilitate inclusive play, children’s preferences, parental inexperience, and inappropriate technology.

### 3.2 Design and Evaluation of Incloodle 1.0

Drawing on these findings from our prior formative study, we designed and developed *Incloodle 1.0*, a picture-taking tablet application for two children to collaboratively play together on a single iPad device [79]. We chose to design for a touchscreen tablet specifically because of the device’s popularity, portability, and usability among children [1, 72]. We focused on using the camera because photography can trigger conversation, contextualize experiences, and serve as a learning tool for social and emotional development [15]. Additionally, Incloodle 1.0 introduced different social and emotional learning topics through character anecdotes, questions about the children, and prompts for the children to take pictures together that correspond to each topic. The topics included subjects such as happiness, fear, embarrassment, silliness, and cheering up others. We developed and curated the topics and the wording of the written and spoken questions and prompts based on our formative study [78], children’s literature that focuses on social and emotional learning and teaching about disability [8], and help from an early childhood education teacher.

We evaluated Incloodle 1.0 in a mixed-methods 2 × 2 within-subjects laboratory study, where eight pairs of neurodivergent and neurotypical children (age 4–7) played with four versions of the application. These versions varied in terms of whether or not the app technologically enforced cooperation between children during joint picture-taking and whether or not characters prompted children to take pictures together [79]. Our analysis revealed that technology-enforced cooperation helped children take pictures together when they had a difficult time cooperating without enforcement. Yet, for the times when pairs did not need enforcement to cooperate, they did not need the enforced rules too. Additionally, the involvement of characters in the application did not have substantial quantitative or qualitative effects on inclusive play between the dyads of children in this short-term lab study. Still, tapping buttons to perform actions within Incloodle 1.0 provided an opportunity for the children to practice turn-taking. The study also highlighted the potential for interactive picture-taking of objects and faces with review of those pictures as a successful way to facilitate inclusive play in that children copied each other’s picture poses and also cooperatively reviewed their pictures together, including mentioning, pointing, and laughing at their funny faces.

### 3.3 Design of Incloodle-Classroom

The results of the lab-based evaluation of Incloodle 1.0 indicated the impact particular features of the application had on neurodiverse pairs of children when they played with it together in a controlled setting. For neurodiverse pairs to use the system in a natural context, we needed to adjust aspects of Incloodle 1.0’s technological enforcement and character-based content, in addition to iterating on the design of the application as a whole to sustain children’s longer-term engagement.

**Fig. 1.**
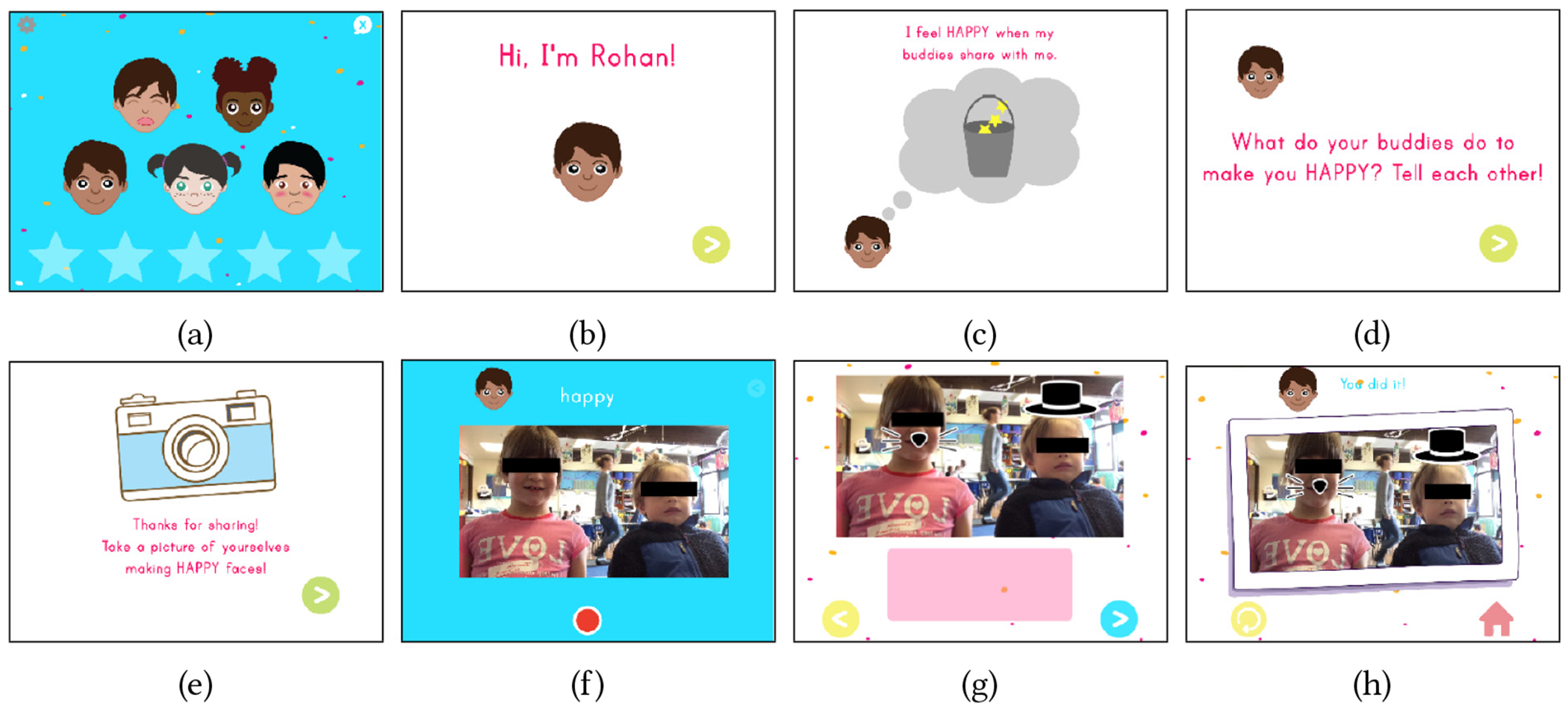
Incloodle-classroom main play screenshots, ordered chronologically, left to right, top to bottom. Original character images by ©Liz Aragon. Updated characters by Lucas Colusso and Mackenna Lees.

In *Incloodle-Classroom*, each child has their own “account” that the child or an adult can choose from the start page of the application. When two children have been chosen, they can enter into the main screen to begin playing (see Figure 1). From this main screen, there are five characters to “meet” (Figure 1(a)) who, when chosen, tell the playmates anecdotes about themselves and prompt the children to talk to each other about different social and emotional learning topics (Figure 1(b)–(d)). The children then take pictures of themselves together making faces or with objects that relate to the topics (Figure 1(e) and (f)).

Afterwards, they can decorate their pictures with stickers (Figure 1(g) and (h)). Each time the playmates “meet” a character, it moves down to a star at the bottom of the screen (to show the play progress) and is replaced by a new character (Figure 1(a)). Once the playmates “meet” five characters and consequently take five pictures together, the five stars of the progress bar fill up. The children can then select one picture among the five that have been taken. The selected picture is shown enlarged. The children can go back to re-select a different picture or proceed to print two copies of the selected picture to a wireless (AirPrint) printer for each of them to keep.

In relation to technology-enforced cooperation for picture-taking, we implemented a toggle that allows an adult to turn on or off the enforcement from a settings screen. Here, the adult can choose whether or not a pair of children need this type of embedded support during their play. Concerning character-based prompting and content, with the help of two other designers, we updated Incloodle 1.0’s characters and developed new ones, resulting in 25 total characters (See Table 1 in the Appendix for a complete list of the characters and stories in Incloodle-Classroom).

**Fig. 2.**
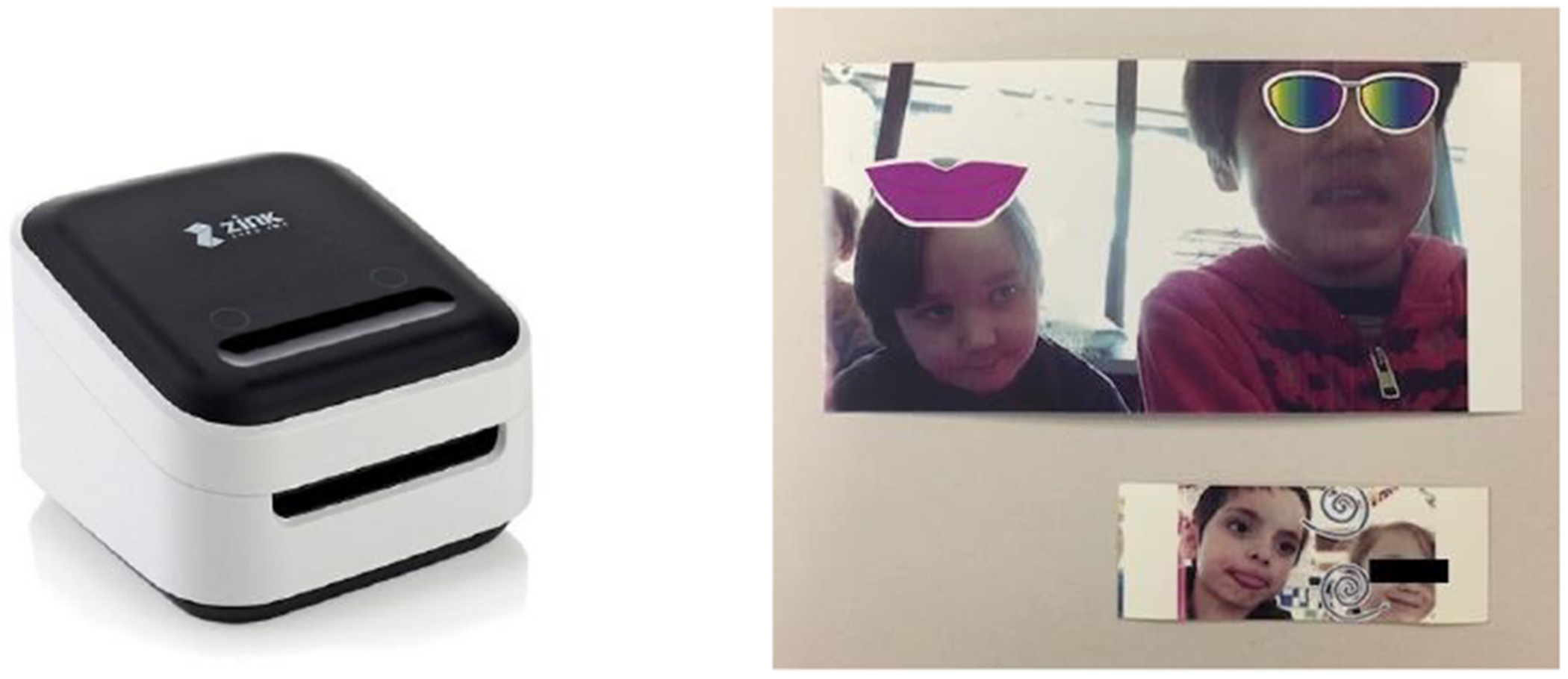
Left: ZINK hAppy printer. Right: Examples of ZINK printer photos.

Incloodle-Classroom is implemented in Swift 3 within Xcode 8.3.3. The application operated on an iPad Air 1, running iOS 10.3. The wirelessly connected printer was a ZINK hAppy printer which does not require ink cartridges, and prints pictures as stickers on rolls of ZINK (or zero ink) printer article, ranging from 0.5″ to 2″ in width (Figure 2).

## 4 Methods

### 4.1 Participants and Setting

Over the course of ten weeks (January–April 2018), the first author (Sobel) brought the *Incloodle-Classroom* app twice per week to two combined inclusive kindergarten classes that consisted of 31 students. Fifteen of these students had **individualized education programs (IEPs)**^3^ and were diagnosed with disabilities, such as autism, developmental delays, and “*other health impairments*.” All 15 of these students are considered to be neurodivergent while the rest were neurotypical. This classroom focused on successful inclusion as discussed in Section 2.1.2, largely following Building Blocks of Sandall et al. [74] (see [78] for more detail). In addition to teachers supporting students individually with their IEP goals, the classrooms focused explicitly on social-emotional learning one day per week, during which neurodiverse children were paired up to cooperate and play at different stations. Through the deployment of Incloodle-Classroom, we aimed to align with the classroom’s existing pedagogical practices and to support movement toward successful inclusion.

Play sessions with Incloodle occurred during free play time in the classroom and lasted about 45–60 minutes. During the sessions, pairs or triads of children (and sometimes more) played with Incloodle on an iPad with a child-friendly case while sitting at a table. The iPad was positioned on a stand to ensure that children had their hands free, as opposed to holding the tablet or worrying about tilting it to a particular angle to capture their faces. Groups of children played with Incloodle for as long as they desired and/or up until they were able to print a picture (usually 8–15 minutes). Some children played for as long as 20 minutes or for as short as four minutes (i.e., stopping before getting to print pictures). Thus, events—or “stretches of interaction that cohere in some manner that is meaningful to the participants” [48, p. 57]—occurred from a group’s start of play with Incloodle to their end of play, which was usually designated by the children receiving their printed pictures.

At the beginning of the intervention, children were asked to play with Incloodle in assigned pairings/triads by teachers in a certain order and then children assented to participate. After almost all of the children had a chance to play with Incloodle using this method, children “signed up” to play with Incloodle as a personal choice, unless teachers personally requested they participate. In this case, a teacher might request particular children/pairs participate because playing with Incloodle was somehow tied to a child’s or children’s play plan or a child’s individual goals. At this point, children were either able to pick their own play partners, or they were assigned “buddy pairs” or “buddy groups” already for the day and played with the app in this same pairing or group. Due to this variable process of choosing/assigning playmates, children were not always in neurodiverse pairs or groups when co-playing with Incloodle.

Teachers in the classroom sometimes, yet much more rarely due to their other responsibilities in the class, interacted with or helped the children while they were playing with Incloodle. Rather, as someone who had volunteered in this classroom in prior years and knew the teachers from this experience, Sobel was trusted with and expected to facilitate the children’s play. Therefore, in addition to the children and teachers in the classroom, Sobel is also an interactant in many of these sessions and, hence, also part of the socio-material context.

### 4.2 Data Collection

Two video cameras recorded the children playing with Incloodle, which started and stopped with each play event. This resulted in a total of 84 distinct recordings of play events, totaling about 837 minutes or 13.95 hours of video data. One camera captured the children’s faces and one captured their backs with some view of the tablet screen.^4^ The app also saved every picture the children took together during these play events onto the tablet’s camera roll (as seen in Figure 1(f)–(g)).

As play with Incloodle happened in the same way that typical technology-based activities occurred in the classroom (i.e., students already played with iPads in the classroom), as per the Institutional Review Board and the school, parents did not have to consent for their children to participate in this study. In this way, all children in the classroom had the same opportunities to play with the tablet, with their assent. However, we sent home information sheets about the study to parents with our contact information. We also included a media consent form, which parents could send back signed to provide permission for their children’s images to be used in public presentations and publications. As a token of appreciation for their children’s participation, at the end of the study, we sent home thank you notes and copies of the book *We’re Amazing 1, 2, 3!* ^5^ with every child in the classroom.

### 4.3 Data Analysis

Our analysis follows multimodal interaction analysis [64] and video research in the learning sciences [21]. Our data comprised video, fieldnotes, and memos made during the intervention, and the pictures that the children took together on the tablet’s camera roll. Analysis mainly focused on the 45 (out of 84) play events that involved at least one neurotypical child and at least one neurodivergent child. Among the rest, 24 events only included play between two or more neurotypical children; and 15 only included play between two or more neurodivergent children.

Two researchers began by content logging the play events in which at least one neurodivergent and one neurotypical child played with Incloodle together. Then, we took a grounded theory approach to open coding our content logs [18], discussing and revising the codes throughout the analysis until we came to consensus. After each coding half of the content logs, we reviewed and updated each other’s analyses. During this process, we also utilized the fieldnotes and the camera roll pictures to supplement our evolving arguments, either matching or contrasting with codes and data. This constant comparative process resulted in 55 sub-codes, which we organized into six overarching codes: adult involvement, equitable participation, inequitable participation, verbal communication, reflection/connections, and positioning for inclusion/exclusion. While content logging, coding the data, and organizing our sub-codes, we made analytical notes in the content logs identifying “hotspots” or salient interaction sequences.

We then transcribed these sequences, which were strategically selected for deeper analyses to identify and examine consistent and contrasting patterns [21], reflecting on the fact that transcription is underpinned by our theoretical foundations, guiding research questions, and goals [65]. Our transcripts integrate verbal and non-verbal interactions and include as interactants not only the human participants but Incloodle as well. Additionally, we utilize the transcription conventions from [47] (see Table 2 in the Appendix for specific conventions used). We also include snapshots of the interactions into the transcripts to display multimodal interactional patterns and embodied communicative modes [64]. Besides those of the researchers, all names in the transcripts and in the discussion of the interaction sequences are pseudonyms.

Throughout logging, transcription, and analyses, we focused on child-Incloodle/Incloodle-child, child-child, and adult-child/child-adult interactions (e.g., orientation, posture, gesture, talk, and so on) as analytical units. In this way, we examined participation structures—or “fluid structures of mutual engagement and disengagement,” characterized by body positioning, eye contact, tone of voice, and other situationally appropriate resources [48, p. 67]—in relation to the designed technology. Centering around these participation structures allowed us to study “the C-issues” which Jordan and Henderson [48, p. 69] refer as “cooperation, conflict, conviviality, competition, collaboration, commitment, caution, control, coercion, coordination, co-optation, combat, and so on,” and are significant in regard to equity and inclusivity. In the findings section that follows, we examine a series of illustrative interaction sequences to exemplify how Incloodle consistently structured children’s inclusive play, shaping what it meant to be inclusive (or exclusive) and equitable (or inequitable) in this socio-material context. All sequences presented below depict interactions between a neurodivergent and a neurotypical child, except one sequence that involves two neurotypical children talking about their neurodivergent classmates while playing with Incloodle.

### 4.4 Positionality and Reflexivity

We frame our research through a Disability Studies lens [9, 41, 52] that cautions against creating technologies from an “ableist view” by attempting to “fix” disabled individuals or make them communicate, act, behave, or think like non-disabled people. Instead of only focusing on therapeutic, interventionist, and pragmatic uses of technology for neurodivergent people, with this non-reductionist view, technology can scaffold, mediate, and enhance interactions among disabled and non-disabled individuals [10, 41]. Additionally, we take an integrative exploratory approach to our research following [29, 69]. We designed for inclusive play considering the experiences of multiple actors, and engaged in constant reflection in this multifaceted and complex situation. We began our research by exploring the design space and iteratively designing while documenting and reflecting on the process. We carried out a field intervention with children with and without disabilities since our design must address the needs of both play partners (i.e., what Pullin [69] calls “resonant design”). Ultimately, using an integrative methodology prompted us to think beyond one perspective [29, 69] and explore this design space from complementary angles.

Our analysis is also guided by our 5-year long partnership with the school, including Sobel’s volunteer work as a teachers’ assistant for approximately 70 hours over eight months at the kindergarten classroom [78]. Sobel’s immersion at the fieldsite provided deeper insight into how this classroom promotes inclusive education and social and emotional learning among neurodiverse children. Nevertheless, we acknowledge that our position as neurotypical researchers inherently shapes our analytic process in which we design and conduct the study and interpret data [88].

## 5 Findings

Below we break down our findings according to six significant themes, which we describe using a narrative-oriented approach with illustrative transcripts [21]. We begin by explaining how the characters’ stories in the Incloodle-Classroom app could be resources for reflection on and connection to children’s own qualities or qualities of their classmates. We then explain how children followed or did not follow the prescribed structure of Incloodle, impacting their interactions with each other and with Incloodle. Next, we demonstrate the ways in which children negotiated their physical and virtual space, as constrained by Incloodle’s camera view, and subsequently we discuss how adults are intertwined in the socio-material context of inclusive play with the app. Finally, we describe how printing pictures was significant to and reinforcing for children, and we show how pictures became “proof” of inclusion (or exclusion), sometimes distinct from the interactions that happened behind the picture.

### 5.1 Connections and Reflections on Children’s Characteristics and Experiences

In our laboratory study with Incloodle 1.0 [79], the characters, their anecdotes, and prompting did not have a significant positive influence on children and their interactions with each other, nor their engagement with the application. Yet, in this setting in which the children were more familiar with each other, the characters in Incloodle provided interactional space for the children to think and talk about the social-emotional content presented as it related to their own or each other’s lives. Not every character or story was successful in prompting every pair to reflect or connect on social-emotional topics; in fact, the most answered questions were those with more straight-forward answers, unrelated to particular emotions (e.g., What are your favorite colors? What are your favorite animals? What are your favorite numbers?). However, in the instances where this deeper reflection did happen, children had poignant verbal answers to questions and displayed meaningful, embodied understandings of particular emotions in their pictures.

In a key interaction sequence between Anna (neurotypical) and Russell (neurodivergent), Incloodle’s character Charlotte presents a story about what makes her feel left out, and then asks the children to talk about what makes them feel left out (Transcript 1, line 1). Anna ignores the question and begins making a funny face toward the tablet (line 2). In contrast, Russell takes a moment to think about the question and answers a related question about what makes him sad (line 3), while Anna looks at him. Looking back at the screen, Anna responds to Russell to say she also feels sad in the same situation (line 4). As Incloodle goes on to prompt the children to take a picture of themselves, Russell ignores the audio and goes on further to explain what happens when he feels sad (line 7). Anna, who stops herself from going to the next screen, looks to Russell and listens (line 6). While pulling his shirt up to his chin over his mouth, Russell explains he covers himself up when he feels sad: “*I make myself INSIDE me*.” After facing toward the camera screen, Anna and Russell look at themselves through the screen for a second and then Russell puts his shirt up over his face (line 8). Anna sees what Russell is doing through the camera view onscreen, rather than by looking directly at him. She smiles and copies Russell by putting her shirt up over her face for the picture (line 9; resulting picture on line 11).

Here, we see two important yet contrasting interactions from the two children. Russell uses the prompts to reflect on his own emotions and experiences and explains and shows what he does (perhaps both metaphorically and physically) when he feels sad to Anna. During this time, he stands and is physically positioned toward Anna. Moving toward the screen, Russell then uses his embodied emotion of hiding for the “*feeling left out face*” picture. At the same time, Anna is less engaged with the question posed by Incloodle yet also more often oriented toward the tablet, as opposed to physically positioned toward Russell while he talks. Still, Anna consistently interrupts herself from interacting with the app to listen to Russell. She hears Russell’s answer and agrees (“*Me too*”), and when actually seeing Russell’s pose for the picture through the camera, she copies it, displaying an embodied understanding of Russell’s experience through her mimicking. In this interaction sequence, Incloodle acts as a resource for inclusive play, prompting reflections for Russell, inhibitory control and empathy for Anna, and a joint embodied understanding of sadness through picture taking for both children.

In another play event between two neurotypical children (Transcript 2), Geoff and Vanessa, Incloodle takes up a similar role, providing a basis for reflection and connections to, in this case, some of their neurodivergent classmates. After Incloodle’s character Lexi says that she feels angry when sounds are too loud because they hurt her ears (line 1), Geoff turns his head swiftly toward Sobel and excitedly exclaims that his classmate Kevin (who is neurodivergent) feels angry in the same situation (line 2). When Sobel responds by saying, “*Oh, yeah?*” (line 4), both children concur by saying “*yeah*” (line 5–6). Even after they move onto the next screen, Geoff thinks of and enthusiastically tells Sobel about another (neurodivergent) classmate Andy (line 9) who has a similar sensitivity to sound (in fact, Andy often wore noise-canceling headphones in the classroom).

While neither Geoff nor Vanessa answers Incloodle’s question or otherwise engages with each other in relation to the question about themselves, Geoff relays information that he knows about his classmates’ sound sensitivities, to which Vanessa agrees. Thus, rather than (only) prompting reflection on their own feelings or experiences, Incloodle allows children to associate what they know about their friends to the character within the app. This is an important evidence that the character content, though somewhat simple in graphical representation and with short anecdotes, can provide learning opportunities and connections between children’s knowledge about the diverse experiences, abilities, and needs of the people around them to what is presented in the app.

### 5.2 Making Sense of and (Not) Following Incloodle’s Prompts

In the laboratory study with Incloodle 1.0 [79], child pairs either followed the prompts of the app to take pictures making certain faces or with certain objects or they did not. Sometimes, when children did not follow the “rules” of the picture-taking prompts, they had more fun; they covered up the camera, did not put their bodies in the picture, or deliberately made faces that did not match what the app was telling them to do. In the case of the current study, children had the chance to play with Incloodle multiple times over multiple weeks, learning about how the app worked and demonstrating their understanding of what they were supposed to do or what they wanted to do, in line with or despite the prompts (e.g., through dominating interactions and/or trying to teach their play partners). In this way, Incloodle became a semiotic resource that gave both interactional rules and goals for children and elicited subsequent responses from and interactions between children.

For example, in Transcript 3, Incloodle’s character Ashley prompts Lisa (neurotypical) and Adam (neurodivergent) to take a picture with a circle (line 1). As Adam had played with Incloodle in the past, he knows through this play that the app (or the researcher i.e., Sobel) wants the play partners to take pictures with objects that match the prompt. Adam gasps, stands up, and tries to explain to Lisa that they need to find circles to put into the pictures (lines 3, line 5), yet Lisa, who is the more dominant driver of the interaction with Incloodle, is not convinced. She does not change her positioning (line 4) until Adam begins to walk away (line 5). At this point, she grabs Adam’s arm to bring him back to the app to take the picture, essentially demanding that he do so (line 6). While Adam continues to stand, Sobel intervenes to explain Adam’s line of action (line 9), which gives way to Adam being able to leave to find a circle (line 10). Not until Sobel questions Lisa about finding a circle (line 11) does she respond. Still, she decides she wants to find something of a *different* shape, i.e., her favorite shape (as she explains earlier), a heart (line 12).

During this interaction sequence, Lisa and Adam go back and forth, verbally and nonverbally, around the meaning they gathered from Incloodle’s directions, whether informed by prior play (most likely the case for Adam) or perhaps ignored, misunderstood, or not heard at all (which may be the case for Lisa). Lisa asserts her dominance over the interaction with the app and with Adam by physically moving him back to the interaction space, while Sobel intervenes to explain that Adam is “right” (or at least warranted) in his request/actions. Having already tried to tell Lisa about what they needed to do next—following the structure of the application and attempting to enforce that structure, Adam is ready to move on with his goal of finding a circle, walking away in two instances (line 6, line 10) when Lisa does not follow his lead. Yet, even after Sobel specifically prompts Lisa to do what Incloodle asked, Lisa reinterprets the task to fit her own idea of what the task should be.

Similarly, in Transcript 4, Incloodle’s character Maya prompts Tristan (neurotypical) and Jeremy (neurodivergent) to take a picture of themselves according to a particular directive—in this case, making grumpy faces (line 1). Jeremy begins making his own grumpy face (lines 2, 5) before the camera screen even appears; he keeps his mouth open, clenches his teeth, and slightly curls his upper lip. When Tristan gets under Jeremy’s finger to press the next button, Jeremy’s grumpy face subsides (line 7). Then, Tristan provides a model of what grumpy means here (line 7)—he opens his mouth to show his teeth, furrows his brow, and growls aloud. Jeremy copies his playmate, making the same face and growling in the same way (line 8). Tristan presses the button to take the picture, again under Jeremy’s finger, and the two playmates smile at the resulting picture (lines 11–12).

Unlike the interaction sequence of Lisa and Adam in Transcript 3, the mirrored yet parallel actions of Tristan and Jeremy in relation to what Incloodle presents are fluid and embodied. Neither child explicitly or verbally states how they make sense of the prompt of the app nor do they position themselves or look toward each other at all. Instead, they follow the instructions side-by-side with Jeremy ultimately copying the (inter)actions of Tristan with the app. They engage with each other and with Incloodle in a collaborative way, while at the same time not necessarily cooperating with button pressing (lines 5–6, lines 7–9). Incloodle acts as a mediational tool and resource for Tristan and Jeremy, allowing them to equitably participate with each other *through* the joint interactional space around the app without having to directly interact with each other.

### 5.3 Negotiation of Physical and Virtual Space

Parallel to how the text and verbal prompts in Incloodle provided meaning, structure, and interactional space for engagement, the camera view in the app structured and impacted how children interacted when taking pictures together. Since Incloodle limited the space in which they could be “seen,” children had to negotiate both their physical and virtual space, as they, the app, or Sobel attempted to physically include them in the picture.

Before the interaction sequence of Transcript 5, Lisa and Adam were prompted by Incloodle’s character Ashley to take a picture of themselves with a circle (Transcript 3, line 1). After “finding” what they wanted to put into the picture (i.e., Adam’s circular object and Lisa’s hand heart gesture) (as seen in Transcript 3), Adam first positions himself in front of the tablet, and afterward Lisa walks up to the Incloodle app. Lisa immediately pulls down Adam’s hand holding the circular object (Transcript 5, line 1) to make room for herself and her heart gesture. She positions her hands so that they are captured by the iPad camera (line 3); however, at this point, Adam and his circle are no longer included in the picture. Sobel takes notice of this and asks Adam if he wants to put his circle back into the picture (line 5). Following, he positions his circle back toward the iPad (line 6) but, in turn, blocks Lisa and her heart. Due to this obstruction, Lisa announces that she is being blocked and pushes Adam’s arm out of the way (line 8). Sobel intervenes to position Adam’s hand in such a way that would include his circle, Lisa, and her heart hands (line 9–12) and press the shutter button for them (line 13).

Here, we see a negotiation around physical space as dictated by what is included virtually in the picture. Both children try to *include themselves* and/or their objects into the picture itself yet, at least initially, struggle to make physical room for each other that would be reflected in the virtual space. It is also apparent in this interaction sequence that Sobel, while not explicitly intended through our design process, became an active part of the Incloodle system. In this case, Incloodle dictated a goal—take a picture—which the children followed. Positioning in the real world became complicated and constrained as it was structured through the lens.

Comparatively, Transcript 6 presents a different instance of physical and virtual space negotiation. Here the interaction sequence involves one neurotypical child, Sam, working to orient the camera to include his neurodivergent play partner, Howie, which contrasts the struggle between Lisa and Adam in Transcript 5. After Incloodle’s character Joey prompts the children (Transcript 6, line 1), Sam presses the button to the next screen where he and his play partner are supposed to take a picture making loving faces. For three seconds, he hovers his finger over the shutter button, waiting while both of the children stay in place to take the picture (line 3). Howie does not make any acts to move or interact with Sam nor Incloodle during this time. Through closer examination, one can see that the camera view only includes half of Howie’s face in the picture (line 3); consequently, Sam pulls his hand away from the tablet (line 5) and tries to turn it toward Howie (lines 6). When the iPad almost falls off the stand, Sobel steps in. At other times, Sam leads the interaction, pressing the shutter button and successfully taking a picture that includes both him and his play partner.

Again, in this sequence, we see a change in physical positioning to accommodate virtual inclusion. Yet, the interaction is driven by one play partner, Sam, while the other stays static, without speaking or moving. In this way, Sam becomes responsible for doing the “including” and deems what “counts” as being an appropriate picture for the two of them. Rather than trying to move Howie away, like Lisa did to Adam, he changes the position of the tablet itself, ultimately reorienting the lens to be inclusive of their physicality in the moment.

### 5.4 Adult Scaffolding as Integral to the Incloodle System

While Incloodle gave structure for interactions between children, either through prompting of questions or by providing virtual cooperative space for picture-taking, it was evident that adults (i.e., Sobel or the teachers) needed to support children’s engagements and (inter)actions between each other and with Incloodle in a significant way, particularly due to the diverse abilities and needs of children in conjunction with the open-endedness of the app. This way, Sobel, along with teachers in the classroom, consistently became entangled in the socio-material context of children’s play, as exemplified in Transcript 5.

Following what we know about the benefits of JME and scaffolding [81, 83] and adults’ roles in facilitating inclusive play [16], teachers and Sobel helped in areas where the children needed support—physically positioning the iPad, mediating turn-taking and communication, providing additional positive reinforcement, or otherwise contextualizing their play. This scaffolding was most vital in two different situations. The first was in situations where the neurodivergent child in the pair had goals in their IEP related to specific behaviors (e.g., self-calming, self-regulation, flexibility, asking for help, negotiation, and so on) or communication (e.g., practicing verbal language, expressive non-verbal communication, and/or social non-verbal communication) and already received accommodations in the classroom related to those IEP goals (e.g., clear expectations of behavior, positive reinforcement for appropriate behavior, demonstration of instructions when introducing new tasks or content). In the second situation, the neurotypical child needed support in being more patient with or accommodating of their neurodivergent play partner. This latter situation also matches technology enforcement’s successful role in supporting inclusive play in the lab study with Incloodle 1.0 [79].

An example of the first situation occurs in Transcript 7. Here, Claire (neurotypical) is playing with Gabe, who is non-speaking and currently working on his communicative language. A teacher sits down next to Gabe to assist him in playing while Sobel sits and watches behind Claire. Incloodle’s character Ashley asks the children what their favorite colors are (line 1), and Claire answers, speaking directly to their teacher, rather than to Gabe (line 2). The teacher repeats what Claire says, and prompts them to think about what Gabe’s favorite color could be (line 3). The teacher and Claire enter into a conversation back and forth during which the teacher offers different ideas about what Gabe’s favorite color could be based on what he wears, and Claire looks back and forth from Gabe to the teacher (lines 4–9). Here, we can begin to see the work of the teacher to include Gabe in an inaccessible part of Incloodle; Incloodle prompts the children to talk to each other verbally, not allowing them to use the screen as an interactional space to answer the questions through the application itself. While Claire does not verbally interact with Gabe, she looks at him in the ways that the teacher prompts her to think about Gabe’s favorite color.

When Claire advances them to the next screen, Sobel comes back with a green block that they can use in their picture, attempting to lessen the burden of either child having to get up and interrupt their play (line 12). Again, including him in play, the teacher tells Gabe where to tap on the iPad to get to the camera and demonstrates by pointing (line 15). Gabe follows his guidance (line 16) and then grabs the green block from Sobel’s hand (line 17), which the teacher helps him position in the camera view (line 18). So that the children do not have to move, Sobel reach around Claire and tap the shutter button for them (line 19), and the teacher gives positive reinforcement (line 21). Claire moves stickers onto both her and Gabe’s faces (line 22), blocking her own eye but not blocking Gabe’s face (decorated picture in line 27). Both the teacher and Claire laugh (lines 23–24), and Claire looks at Gabe (line 24). Including Gabe into the interactions, the teacher acknowledges what makes the sticker placement on Gabe’s head funny and physically demonstrates where it is on Gabe’s head (line 25). Claire then points to her own picture to acknowledge why her picture is funny as well (line 26).

Throughout this sequence, Gabe’s inclusion into play with Incloodle would not have been possible without their teacher being involved. Incloodle could have been and still could be more accessible for non-speaking children by allowing them to answer questions physically instead of verbally (e.g., through tapping specific options on the screen; although this may have other accessibility effects). However, the support that the teacher gave Gabe—bringing him into the conversation, prompting his actions onto the screen, physically supporting him during picture taking, and involving him in the picture reflection ensured that Gabe’s participation was inclusive and equitable under the circumstances. At the same time, Claire physically included Gabe in the play, along with the support of the teacher. Although not verbally directing her responses at Gabe, she took turns pressing buttons, took up equal space in the picture, and decorated both of their faces, without covering his face, despite her covering her own.

We can further observe subtle examples of adult support in another play event between Tristan (neurotypical) and Howie (neurodivergent) in Transcript 8. Here, the children collaboratively play and communicate with each other mainly through non-verbal language while also engaging with Incloodle; they mimick each other’s faces during picture taking and smile, look at each other, laugh, and point at their pictures afterward. Yet, there are moments where they need slightly more support in engaging with one another verbally or in taking turns. In one of these instances, Sobel intervenes to ensure that their communicative language and behaviors are directed at and recognized by one another, something that Incloodle does not do. However, in a second instance, Sobel misses an opportunity to enable Howie to engage with Incloodle in the same way that Tristan is doing.

At the start of Transcript 8, Incloodle’s character Barika relays an anecdote about her favorite number (line 1) and Howie tries to engage with Tristan non-verbally by turning and looking at him after smiling and laughing (line 2). Tristan does not acknowledge Howie’s non-verbal bid for attention and goes onto the next screen (lines 2–4). Once Barika prompts the children to tell each other their favorite numbers (line 5), Howie turns to look at Tristan, positioning himself for a conversation (line 6). However, Tristan instead turns away from Howie, toward Sobel (offscreen, watching them) to give his answer, and then orients himself back to the iPad to go to the next screen without interacting with Howie (line 7–8). Sobel notices this and verbally interjects by responding to Tristan that he should tell Howie his answer (line 9). This stops Tristan from moving onto the next screen, and he orients his face and gaze to Howie (lines 10–11). Enthusiastically, Howie answers the question, raising his hands above his head while he says his favorite number (line 12).

Even though Sobel’s comment was to have Tristan actually speak to Howie, Tristan takes this as an opportunity to listen to Howie, who in many other play events does not engage with Incloodle nor his play partners (e.g., Transcript 6). In this way, Sobel’s intervention and Tristan’s subsequent actions enable Howie to have a voice, when his typical behavior is not to speak or interject without verbal or nonverbal indication that it is okay to do so first.

Following this verbal exchange, the children easily collaborate during picture taking through more physical communication—being in close proximity to one another (line 20), making similar faces (lines 18–19), and pointing and laughing at their picture (lines 21–22). However, when they both reach out simultaneously to move stickers onto their picture (lines 23–24), Tristan again dominates the interactions with Incloodle not only by saying “no” to Howie trying to decorate the picture (line 25) but also by grabbing Howie’s wrist and moving Howie’s hand down to the table (line 25). Tristan holds Howie’s hand down for the entire time that he moves both of the stickers onto their faces in the picture (lines 25–27). Off to the side, Sobel fails to notice this interaction (in lines 25–27); she does not intervene to let Howie have a turn in putting a sticker on the picture or to ensure Howie has any say in how his own face is decorated in the picture. Still, the children look at each other and laugh at the decorated picture (line 27–28). Finally, Howie moves his hand away from Tristan, who subsequently lets go (line 28).

These two instances within the same interaction sequence give evidence of how a small interjection can make more likely that a child is being included or participating in play equitably. While Tristan, the more dominant play partner, easily takes control over picture decorating when Sobel is not paying attention, a small comment from Sobel about talking to Howie prompts Tristan to turn and listen to Howie, who is overjoyed to give his answer in this context.

Although Incloodle is purposefully open-ended to allow for flexible adaptation to the needs of different children, there is less technology enforcement in interacting with the app. There is no voice recognition to ensure both children are speaking or touch enforcement to ensure both children are getting a turn to tap the screen. Thus, children who need extra support—whether that be in the form of adults enforcing turn-taking or giving additional prompting and oversight to communicate (verbally or nonverbally)—often cannot co-engage with each other and Incloodle without an adult involved.

### 5.5 Significance of the Process and Product of Printing Pictures

With the design iteration of Incloodle 1.0 to Incloodle-Classroom, we added the ability for children to print pictures, with the intention for this to reward or reinforce children playing together. The goal was for the printing experience and materiality of printed pictures to be meaningful to children. We envisioned that having printed pictures would be a physical emblem of their experience, beyond the pictures within the Incloodle app that do not “live” outside of the device and cannot be shared or taken home. Children’s collaborative interactions with the printer and with the physical pictures that they received after playing with Incloodle provided evidence that this goal was met. However, at the same time, agreeing on one picture to print was not as simple for children as we anticipated.

An interaction sequence between Alex (neurodivergent) and Zoe (neurotypical) shows the significance of both the printing experience and the physical pictures to children (Transcript 9). After Alex and Zoe both immediately agree on the same picture to print, Sobel shows them the printer and tells them the picture they chose will come out of it. Alex asks Sobel if “*kids get to keep*” the picture and Sobel confirms that they do. He tells his playmate Zoe this and asks her if she wants to keep hers, to which she responds affirmatively. This is an indication of the significance of the pictures as materials objects that kids can keep to themselves.

Following this, Sobel explains that the printer is going to print two copies of the picture so that each child can have one. Eagerly, Alex asks Zoe if she is ready for the picture to come out and moves his head down to the printer. He tells Zoe to do so as well multiple times, so she can “*see it better*.” As the picture starts to come out of the printer, the children lift their heads in anticipation, giggling and squealing. “*Look it’s right there!*” exclaims Alex. Once the picture comes out of the printer, both children look at it, smiling and laughing (lines 1–2). Similar interactions between children around the printer and with each other continue while the second picture prints. They look up and down at and into the printer, and Alex excitedly and loudly tells Zoe he can see the second picture coming out of the printer (lines 9–11). Through these interactions, we see evidence of the value of the physical picture, as something the children expectantly wait for and orient to.

Once Zoe gets the second picture from the printer (line 12), Alex and Zoe hold their pictures up next to each other, smiling and looking at them side-by-side (line 14). In this instance, the children’s joint attentive gaze at their pictures shows how the picture is not only meaningful to the children but something that they can hold and reflect on directly. Now that the children are done playing, Zoe walks away from the play area and Alex keenly runs toward his classmates at the back of the room to show them his picture (lines 16–17). Alex calls over Zoe to show another classmate her picture too and then tells one classmate, “*Look what we got!*” (line 17). Again, the picture holds meaning to the children, as something they can share and show off to their friends in the class.

Dylan (neurodivergent) and Geoff (neurotypical) have a similar experience to that of Zoe and Alex when waiting for their pictures to print and reflecting on them (Transcript 11); however, their experience agreeing on a picture to print was not as seamless (Transcript 10). Here, we describe these interactions chronologically from picture selection to printing.

In Transcript 10,^6^ Dylan and Geoff are prompted to pick the picture that they both want to print. Dylan points to one picture he wants to print, which has “*BOTH*” of them in it (line 2, line 6).

However, Geoff disagrees with Dylan on printing that picture (lines 7–13). Instead, he points to a picture that has more of his body/face in it compared to Dylan, which Dylan notices (“*But that only has mostly you in it*.”) (line 15). Geoff responds that he does not care (line 16), and Dylan offers his original picture as an option that includes both of them equally in the picture (lines 17–19).

Rather than attempting to make the children cooperate and come to a shared decision about one picture to print, Sobel tells them that she will print them two copies of both pictures (line 20). Geoff nods to say he is okay with this decision (line 21), and Dylan excitedly exclaims, “*Two of that and two of that!*” while pointing at the pictures (line 22). In this interaction sequence, the picture selection process reveals a tension; the pictures hold meaning to children as something they want, yet they want different pictures for different reasons. In line with the goals of Incloodle, Dylan wants a picture that physically includes both of them in it. Yet, Geoff wants a picture that features more of him in it as opposed to his play partner. While getting a printed picture that they want is important, the fact that the children cannot agree upon one picture and that Geoff’s choice is not inclusive of Dylan reveals a conflict in what Incloodle intends (to promote inclusion) and what a child might want at a particular instance.

A subsequent interaction sequence with Geoff and Dylan further shows the worth that the pictures have for both children, which they recognize as a shared yet distinct interest (Transcript 11). After Sobel exits out of Incloodle to print copies of the two pictures, she asks them if they are done playing and Geoff says he wants “*ours to print, both of ours*” (line 1), reinforcing the importance of the pictures and how they want different pictures. The children run over to the printer to see their pictures print, with Geoff exclaiming, “*I got one! I got one!*” (lines 3–4). Once Geoff gets the picture, he cheers, “*YES!*” but questions why the picture is not the one he chose (lines 6, 8, 10). When Sobel lets him know that that one will come out later, he rejoices (“*YAY!*”) (lines 11–12). At the same time, Dylan queries where his picture is (line 7). When another picture starts to print, he celebrates (“*Mine’s coming!*”) and looks down into the printer (line 13), similar to how Zoe and Alex did. As soon as he gets the picture, he yells (“*Ah!*”) while looking at it (line 15). He jumps up and down excitedly, explaining that the picture he has will be for him and his stuffed animal (i.e., “stuffie”) and physically holds the picture over the stuffed animal (line 17). As his speech and interaction with the picture and “stuffie” show, the picture becomes something he can keep and share with other objects that are meaningful to him.

Later, Geoff says that he gets the next picture and Dylan responds by saying he gets the “*OTHER next one*” (lines 18–19). Here, the children are simultaneously interested in getting and keeping their pictures; however, for Geoff, this is more about receiving the picture that he specifically wanted, contrasting Dylan, who is excited because they both have pictures.

In the cases of Zoe/Alex and Dylan/Geoff, we see the printer and pictures as motivating. The printing experiences and the pictures are central to their interactions, both speech and movement, portraying anticipation and excitement. The children are also enthusiastic by the prospect of keeping and sharing their photos. Alex runs and shows his picture to a classmate named Faith, calling Zoe along, while Dylan exclaims how his picture is going to be for him and the stuffed animal he made. Nevertheless, while Zoe’s and Alex’s interactions around pictures and printing involved one picture they both easily chose, the picture selection experience for Dylan and Geoff was not as easy. Although they were jointly attending to the printer and pictures, Geoff’s interest in having a separate picture from Dylan shows how the picture itself does not necessarily hold meaning around inclusion and cooperation for children in the way that we embedded it into Incloodle.

### 5.6 Pictures as a “Proof” of Inclusion vs. the “Work” of Inclusion Behind the Picture

Analysis of the pictures saved to Incloodle’s camera roll in conjunction with the collected video data revealed another tension between what was captured by Incloodle—a frozen moment of interaction in the play event—and what was captured via video recording—the interactions actually involved in that play event. Transcripts 12 and 13 present two narratives in which the picture could become proof of inclusion (or exclusion) during play with Incloodle, despite the actual interactions that occurred to achieve the picture.

In Transcript 12, while not fully visible in the recording, Sobel can be heard off-camera, as she notices Russell (neurodivergent) leaning in, covering the lens, and blocking Zoe (neurotypical) from being seen in the picture. Sobel quickly intervenes, disrupting their play by pulling the iPad off the stand, causing Russell to pull back his face and reorient himself further away from the iPad. Sobel puts the iPad back down, pushing it and the stand further back on the table, widening the angle of view of the lens to include both Zoe and Russell’s bodies and faces. The ensuing picture they take is shown in line 4. However, this image alone, a picture that can be printed, shown to others in the classroom, and taken home, is at odds with the work of inclusion that happened “behind” it. While Sobel structured the picture by moving the iPad, she remains almost entirely invisible in the “proof” of participation, engagement, and inclusion that Incloodle produces.

As this dynamic (of Russell getting too close to the screen and excluding Zoe from being in the actual picture) continued to occur, Sobel turned on technology enforcement. However, this did not stop him from putting his face too close to the camera. Rather, it just prevented him from taking the picture when his face was in that position. In these situations, Sobel still had to intervene and remind Russell that it would not take the picture unless he backed up and included both his and Zoe’s face in the camera.

Similarly, in Transcript 13, Incloodle’s character Nikki prompts the children to take a picture together making cheerful faces (line 1). Zoe (neurotypical) and David (neurodivergent) take turns pressing the buttons (Zoe, line 2 and David, line 6). David counts to three to take the picture together (lines 5–6) while Zoe smiles (line 4). This co-engagement is joyful, involving laughs and smiles, and coordination of body positioning and virtual space in the picture. Yet, when the resulting picture (line 9) is shown on-screen, Sobel calls David “silly” for not being “in” the picture (line 8).

This interaction sequence parallels other instances in which teachers in the classroom had Sobel reprint pictures (including for David) when a picture may not have included a face in it (even if it was the child’s decision). In fact, David often only engaged with Incloodle during the picture-taking portions of the design, running away during the character and verbal discussion portions of the application and then joyously returning to take pictures, counting to three and pressing the shutter button, often not caring if his face was in the picture or not. In this way, Incloodle supported David in equitable, intermittent yet fluid participation with the application and his peers, but this type of engagement was not consistently recognized as being collaborative enough for adults, including the researcher (i.e., Sobel herself), at the moment.

## 6 Discussion

Incloodle-Classroom, a technology that mediates co-participation and collaborative engagement—works within a complex system of (inter)action with socio-material resources. This system complicates what it means to be “inclusive,” depending on those involved (including their abilities and needs), the adult facilitators (when they intervene and what they “count” as inclusive), and what the technology itself engenders/restricts based on what may be more normative, exogenous notions of “cooperation” and “inclusion” embedded in that interactive object.

During our research, Incloodle became a coordinative artifact that allowed us to ask and answer questions about what design is doing to facilitate equitable, participatory collaborative engagement and interactions for children with diverse abilities and needs. Reflecting on our iterative design of Incloodle, taking it into an inclusive kindergarten class over a 10-week period, and conducting a deep analysis of children’s interactions with and around the application, we discuss specific design features and their ability to support children during inclusive play. Drawing on this, we introduce the concept of *inclusive JME* that can offer insights into a future research and design agenda based on our design work and studies on an inclusive play technology.

### 6.1 Implications of Children’s Experiences with Incloodle-Classroom

Through our analytic process, we found Incloodle provided semiotic structure for reflecting on social and emotional topics and for picture-taking activities among the children, whether that be with objects or by making certain faces. The children made connections to their own experiences and the experiences of their classmates, as they related to the characters in the application and the social-emotional topics they presented. They also interpreted directives for picture-taking as necessary or unnecessary by using them to make arguments for what should (or should not) occur in a picture in verbal or embodied ways. Likewise, Incloodle structured children’s participation as they negotiated their physical space to match the structure of Incloodle’s digital space. This was true even in the contrasting cases of Lisa/Adam (Transcript 5) and Sam/Howie (Transcript 6), in which the more dominant drivers of the interactions (Lisa and Sam) approached this shifting of space very differently. However, the ways that these more dominant interactants positioned and moved themselves, their playmate, and the iPad showed the potential for the lens to assemble how children make sense of what counts as engagement (or individual participation) vs. co-engagement (or inclusion or cooperative participation).

Analyses of children’s interactions with each other and Incloodle revealed that teachers and the researcher (i.e., Sobel), as adults, became integral to the Incloodle system as additional mediators of children’s collaborative participation in this socio-material context. We contextualized and scaffolded children’s play, especially by ensuring that neurodivergent children were included in engagements with Incloodle. As in the case of Gabe and Claire (Transcript 7), this involved providing further supports for the neurodivergent child to physically interact with the tablet and making sure that the neurodivergent child was involved in social-emotional conversation and picture reflection. Akin to the case of Howie and Tristan (Transcript 8), this also involved interjecting to remind neurotypical children to interact and cooperate with their neurodivergent playmates.

Children’s interactions with and around the printer and their printed pictures showed the significance of printing to children, not only as a reinforcement for their experience (i.e., a “reward” that they received after playing together) but also as a material artifact that they could share with each other, take home, and reflect on later. Children gathered around the printer, speculating on how it was working and negotiating how they were going to get those pictures. Children also chose pictures to print that included all of their faces and showed them to other teachers and students enthusiastically. Selecting a picture to print, however, was not always easy for the children, as they often disagreed on which picture they wanted to both have copies of.

At the same time, analysis of the resultant pictures of Incloodle problematized the notion of how inclusion was embedded in Incloodle (and by the researcher i.e., Sobel). Therefore, it is significant to think about how Sobel both designed Incloodle and structured co-play with Incloodle—particularly around the picture-taking experiences of the application, as aligning with more exogenous notions of what “counts” as inclusive participation (i.e., [80]). By focusing on the end result (the picture) as proof of inclusive participation, Sobel excluded *the interactions themselves* as having meaning, which may not have always aligned with having a face in a picture. Rather, this presentation of a moment could and did easily become a misrepresentation of children’s equitable engagement and, potentially, endogenous notions of what equitable participation and inclusion could entail for and mean to children, particularly those with different abilities and needs.

Similar to how video recording is itself theory [39], Incloodle’s pictures change what others may or can interpret from those pictures. Even more, like how “[a] recording first and a transcript later should not be presented or seen as attempt to reproduce the entire original experience” [23, p. 308], pictures with Incloodle cannot be seen as the entire original experience; they are only a particular slice *of a particular slice of the universe* in which Sobel was interested. However, these pictures live on, beyond the boundaries of our work, with the children both in the classroom and outside of it, as a new semiotic resource for their friends, teachers, and families. More work needs to be done to understand the wider implications of these types of pictures, which standing alone may do work of inclusion for those that are in them and view them.

Ultimately, Incloodle became an important actor within the socio-material context of inclusive play, shaping what it “means” to be inclusive from the perspectives of children, adults, and the technology itself. When Incloodle provided prompts and structure for discussion and picture-taking, children used this as a basis to interact with each other, connect with their playmates and classmates, and negotiate their positioning in the real world to match what was seen in virtual space, including both themselves and their playmates in pictures. This was not always easy for children to do on their own with the application; and, therefore, the teachers and the researcher i.e., Sobel often stepped in to facilitate inclusion in relation to the application, making sure engagement with and around it was equitable for both playmates. Finally, Incloodle itself embedded notions of inclusion within its design by telling children to converse with each other and take pictures together. Utilizing the camera and printer provided material reinforcement for collaboration and cooperation, as children constructed and then later printed an image of themselves together. In many cases, their photography resulted in “inclusive” pictures in which the children were “seen” as meaningfully participating by both being in the picture. Yet, the picture sometimes did not match children’s intentions and interactions behind it—whether that be (1) due to Sobel’s or face detection’s enforcement of inclusion during picture taking, or (2) due to children not wanting to be “in” the picture (e.g., by covering their faces or moving outside the lens).

### 6.2 Designing for Inclusive Play among Neurodiverse Children

Incloodle is a semi-structured application for collaborative play that is intended to allow for adjustability, adaptation, and individualization based on the children whom are playing with it. Incloodle embeds an understanding of the design space of inclusive play from the perspectives of children, parents, and teacher, and specifically works to address all of the considerations that our formative study [78] identified as being important in designing for inclusive play. In relation to inclusive play with technology, we argue that adults in this context can be just as important as the technology itself to scaffold and contextualize interactions among children. In the subsections that follow, we review the ways in which specific design features can be utilized in other technologies for inclusive play among neurodiverse groups of children.

#### 6.2.1 Photography and Physical Pictures as a Medium for Collaborative Play.

We argue that photography and printing physical pictures are something that the human-computer interaction community should focus on more when it relates to designing for collaborative play experiences between young neurodiverse children. Through the picture-taking process, technology offers embedded support for mirroring behaviors, which relates to feelings of closeness in friendship and peer modeling [32, 70, 84]. It also offers embedded support for cooperation in picture-taking, including shared negotiation of physical and virtual space, joint attention, and learning through embodied understandings of social and emotional learning topics. Decoration of pictures, as discussed in our design of Incloodle-Classroom, allows for further cooperation and personalization of the pictures taken as well. However, we also contend that design paradigms of unlimited stickers or stamps (as we observed in the classroom with other apps) can be distracting by leading to repetitive interactions that do not support progression or learning.

Our longer-term intervention also showed that the materiality of pictures can be meaningful to neurodivergent children during play as well. Physical pictures are artifacts that they can keep as their own, unlike digital pictures on a screen or in the cloud. In this way, printing experiences and physical pictures are reinforcing and motivating for children’s inclusive play and also allow them to reflect on their experiences and share a meaningful inclusive shared experience with other people, like their parents, teachers, and other friends. Thus, material pictures have the ability to live on as a frozen moment in time; although, it is possible that this frozen moment does not actually match the interactions behind it (whether equitable or inequitable, participatory or non-participatory, inclusive or exclusive). There is more work to be done to understand the wider implications of this on adult and child perceptions, attitudes, and behaviors and their “rippling” effect [16].

#### 6.2.2 Technology-Enforced Cooperation.

Although informed by related literature, how we designed technology-enforced cooperation for inclusive play was through an exogenous perspective. While during the foundational research behind Incloodle, we took into account the perspectives, attitudes, and experiences of parents, teachers, and children [78], there was no specific investigation in this study into photography in particular. Rather, we used their design intuition and theoretical foundation to move forward in creating a photography-based experience for inclusive play [79]. However, by utilizing face detection for cooperation during photography, we embedded an exogenous notion of what inclusion should be during engagement with Incloodle. As discussed in Section 6.1, photos may or may not be evidence of inclusive interaction between children. Due to this variability, it is important to *not prescribe* the ways that children can interact with technology through enforcement statically. Instead, like the way we incorporated a toggle to turn on or off tech-enforcement for picture-taking, technology-enforcement in other contexts or designs should be dynamic or changeable by people.

Reflexively, we recognize that our own view of inclusion during picture taking was evident in how Sobel personally scaffolded children’s joint photography experiences by making sure that both children were physically in pictures or commenting that it was “*silly*” or that they needed to retake pictures when both of their faces were not in a picture. Thus, adults need to be cognizant of and reflexive about their role as scaffolders of children’s joint inclusive experiences, paying attention to what is equitable participation and engagement depending on the children who are playing.

Moreover, while we designed a toggle for technology-enforcement into Incloodle-Classroom based on the results of the laboratory study [79], this embedded support for collaboration or cooperation needs further contextualization to more thoughtfully facilitate inclusive play and learning. For example, as it stands now in Incloodle with technology-enforcement, children are required to put both of their faces into a picture without any reasoning or contextualization of why this is necessary. Unlike other embedded supports for cooperation, like providing two scissors to three children during an art activity or having children play with a wagon because it cannot be used alone, it is not clear why a picture can only be taken when two faces are in the camera frame. Either, direct supports are necessary to indicate why it is important to include playmates in this context or there needs to be some other contextualizing factor that might require a reason to cooperate (e.g., two children need to be in a picture, smiling, so that they can show how many teeth they have all together). This may be the difference between having children change their prosocial behaviors vs. learn why it is important to share, compromise, and cooperate with other people in a contextualized way.

There are also many other types of cooperation that could be enforced by design that might lower barriers to inclusive play. For example, children struggling with turn-taking and simultaneously touching the application screen demonstrated that this was a challenging aspect of co-engaging with Incloodle. As in the cases of SIDES [67], Zody [13], the Collaborative Puzzle Game [7], and Untangle [45], technology-enforcement for cooperative on-screen interactions via collaborative gestures or other mechanisms could be successful for neurodivergent children. There is room for exploration to understand how different types of joint interactions might be mediated with dynamic enforcement and contextualization, especially on small devices like a tablet. For instance, examining turn-taking with face recognition (i.e., to determine who is holding the device or taking up more interactional space physically) or joint audio recording (e.g., singing, answering questions) with speaker recognition [50] and how these different kinds of tech-enforcement affect inclusive play could be fruitful areas of future investigation. Additionally, more research could be done to examine neurodiverse groups of children’s co-located use of multiple small portable devices, similar to how researchers examined the co-located use of 7″ tablets and mobile phones for learning by young students in classrooms in India [46, 77].

#### 6.2.3 Character Narratives and Discussion Prompts.

Characters with stories, who prompt reflective, social-emotional topic-based conversations or interactions between neurodiverse children are also productive for inclusive play. These types of characters and discussion prompts offer more direct support for inclusion and social-emotional learning; transparency about disability and how we all have similarities and differences; and a focus on children’s interests and strengths, with the goal of helping children get to know each other better, including their likes, dislikes, and needs. This makes sense as researchers have connected parasocial relationships, or meaningful one-sided relationships with characters [43], to fostering learning goals for children [12, 37].

There also need to be ways to answer questions and engage in conversation about topics that do not come more easily to children, for example by building up social-emotional topics from simpler questions to more in-depth ones over time, according to children’s zones of proximal development [83]. Additionally, technologies for inclusive play need to allow for non-verbal responses and interactions to ensure that children with speech impairments or disabilities/disorders that affect verbal communication can still equitably participate.

Over time, characters had more of an impact in our intervention with Incloodle-Classroom compared to the lab study [79], as children got to know the application and each other better over the months they played together (see Section 5.1). These characters caused connections and reflections on their own and each other’s experiences. Drawing on the vast amount of current children’s media—e.g., television [17, 34, 55], digital games (e.g., “Feelings Games”^7^), books [8], and social stories [36]—that focus on social and emotional learning and/or disability awareness, technologies for inclusive play should continue to incorporate characters whose stories prompt learning about our similarities and differences, likes, dislikes, strengths, and needs.

Similarly, it is important to provide diverse representation and diverse experiences into children’s technologies for inclusive play, such that children have media-based characters and stories to which they can personally relate and that show them the diversity of human experience. This is in line with evidence that meaningful media representation impacts children, like empowering them (vs. reinforcing stereotypes); validating their identities; giving them common ground for dialogues and understanding about diversity, prejudice, and/or unique, intersectional identity-based experiences; and allowing them to more effectively learn from the media (e.g., [5, 27, 56, 73, 82]. Following this idea, Sesame Workshop, the nonprofit organization behind the television program Sesame Street, has spearheaded a national initiative called “See Amazing in All Children,” which aims to combat the stigma and isolation experienced by autistic children and their families and to help increase understanding, reduce stigma, and demonstrate the commonalities that children with autism share with all children [2]. As part of this initiative, they introduced Julia, a new Muppet with autism into their television program, and offered a range of videos for children and parents about Julia, autism, and how all children have needs and strengths. Similarly, a grassroots coalition called Kids Inclusive and Diverse Media Action Project^8^ is supporting the creation of diverse and inclusive children’s media via research, design guidelines, and best practices to help content developers. Following these examples, technology for inclusive play requires the incorporation of children with disabilities and with diverse strengths and needs into its content.

### 6.3 Designing for Inclusive Joint Media Engagement

Takeuchi and Stevens [81] describe six conditions that lead to productive JME: (1) mutual engagement, (2) dialogic inquiry, (3) co-creation, (4) boundary crossing, (5) intention to develop, and (6) focus on content, not control. In the case of our work, Incloodle provided the conditions for (1)–(5). Children were motivated to participate, largely due to the picture-taking aspect of Incloodle (1-mutual engagement). They collaborated to make meaning in answering the questions and taking pictures (2-dialogic inquiry). Together, they built joint understandings of each other and of how to play with the application, while at the same time co-constructing photos (3-co-creation). Engagement with Incloodle allowed children to reflect on their past experiences and gave them pictures to reflect on in the future (4-boundary crossing). And, children were driven to continue to play, meeting characters, taking pictures, and “getting stars” until they could receive a picture (5-intention to develop). In terms of (6) focusing on content vs. control, joint interactions with Incloodle were more difficult for the children in our study; while for some pairs/groups of children, turn-taking, cooperation during picture-taking, and picture selection came easily, this was not true for all children who played with Incloodle.

However, in examining these conditions in relation to Incloodle, we suggest that there is more to take into account for productive JME when co-participants are diverse in their physical, cognitive, behavioral, social, or emotional abilities and needs. We refer to this type of shared experiences with media among people with and without disabilities as *inclusive JME*.

Grounded in our investigations, we offer three more conditions for productive *inclusive JME* in particular. First, engagement must be *adaptable* or individualizable to be equitable and inclusive. Often, this means that engagement should be relatively open-ended or that children have opportunities to make meaning out of their participation with media in ways that match their abilities and needs and not in ways that are narrowly imposed by the media’s design. A related positive example of adapting engagement for inclusive, equitable participation is with the location-based mobile game Pokémon GO [76]. With this application, different parents and children with diverse developmental needs and abilities were able to play the game together because of how the device could be shared during gameplay, despite it not being explicitly designed for collaborative use. Second, engagement should be *empowering, allowing children to demonstrate their strengths*. In conjunction with being motivated to engage and intending to grow through participation (i.e., original conditions for productive JME by Takeuchi and Stevens [81]), when children with diverse abilities co-engage with new media, they should feel confident about their participation and be excited to contribute. Third, productive inclusive JME does not occur in a silo—it should *draw on a myriad of interactional resources*, including but not limited to adult support, assistive technologies, and other contextualizing and/or translational tools or scaffolding. Like how JME that allows for boundary crossing across time and space will be productive, inclusive JME that draws on many interactional resources in situ will ensure that children can connect with each other and with the media they are using. In turn, children can interact with and learn from each other both around and through a technological medium. Future research should examine (1) how different types of technologies may already support these three new conditions and (2) alternative ways to consider adaptability, empowerment, and other interactional resources in relation to co-engagement with technology for children with and without disabilities.

### 6.4 Future Directions

Designing equitable, collaborative technology-based play experiences for neurodiverse children requires reflection and thoughtfulness as to how play occurs between children with and without disabilities. Our work begins to disentangle some of these complexities by unpacking how technology can be used a resource within neurodiverse groups of children’s relationships and interactions [48], and can support children in equitable, inclusive, and participatory collaborative play for learning. Based on this, we prompt other researchers and designers to consider how their designs may facilitate or act as barriers to this type of collaborative engagement. How do our ideas about inclusivity and equity get embedded into what we create? How do these artifacts work within the social-material practice of multiple actors, including both people and things? Who or what is “responsible” for doing the work of inclusion in this context? When or in what situations? How might what we create be used in ways we do not intend to aid or inhibit children from equitably engaging, collaborating, learning, and/or participating? What are the impacts of specific design features and their prescribed notions of use? In the future, longitudinal studies should investigate whether and how design interventions like Incloodle might impact neurodiverse children’s interpersonal relationships and subsequent social play activities in the classroom, plus parental perceptions, beyond the deployment period.

Concerning inclusive JME, future work should study co-engagement of children with a wider range of disabilities (i.e., including physical and sensory impairments), with different age groups (i.e., younger and older children), intragenerational experiences (i.e., between or among children with and without disabilities), intergenerational experiences (i.e., between or among children and adults, either of whom have disabilities), and with other technologies that are not necessarily play-based (e.g., co-viewing videos online, pair-programming in blocks-based coding environments, and so on).

Finally, future work in this area would benefit greatly from getting more direct input from children with and without disabilities in the design process. Within HCI, there has been a strong movement toward not only designing for neurodivergent children but also with them (e.g., [11, 26]). In the same way, to better inform the design of technologies for inclusive engagement among children with diverse abilities and needs, we need children with and without disabilities to participate in design alongside adults. Participatory design with children usually involves children who are older than six because designing with younger children is not usually recommended [26]). However, based on our experiences with children in the inclusive classroom, there is room for innovation on new methods that can support designing with younger and older children of diverse abilities and needs together, inclusively.

## 7 Conclusion

Through our analysis of illustrative interaction sequences as narrative cases, we contribute a generative, empirical understanding of how neurodiverse groups of children jointly engage and play with a tablet application in a natural setting over an extended period of time. We observe that *Incloodle-Classroom*—a technology designed for inclusive play—acts as a coordinative artifact for equitable participation and engagement, and social-emotional learning as a member’s phenomenon [80]. By interacting through and around Incloodle with adult support, children had opportunities to learn about and connect to each other socially. In this way, the interactions that emerged were evidence of children’s joint learning of how to be inclusive spatially, in verbal and nonverbal communication, and in engagement with, around, and through the device.

## Figures and Tables

**Transcript 1.** Anna (neurotypical) and Russell (neurodivergent) talk about feeling left out.

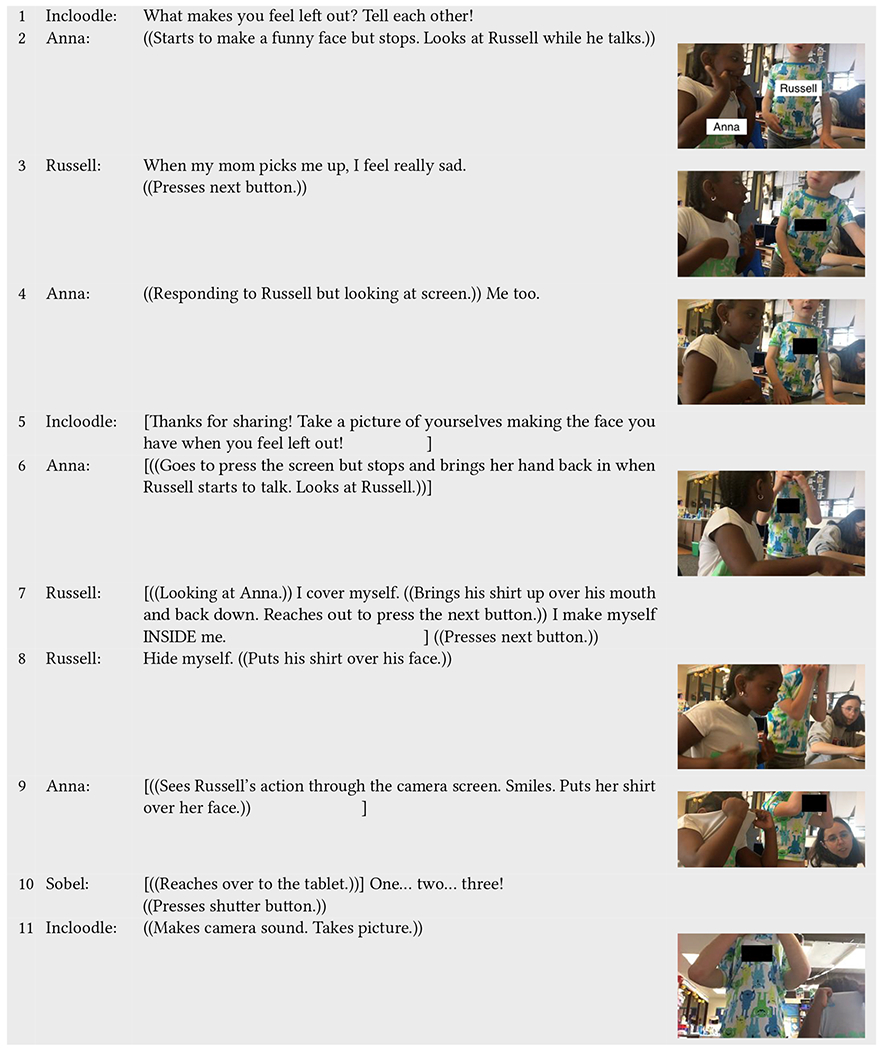

**Transcript 2.** Neurotypical children Geoff and Vanessa talk about their neurodivergent classmates.

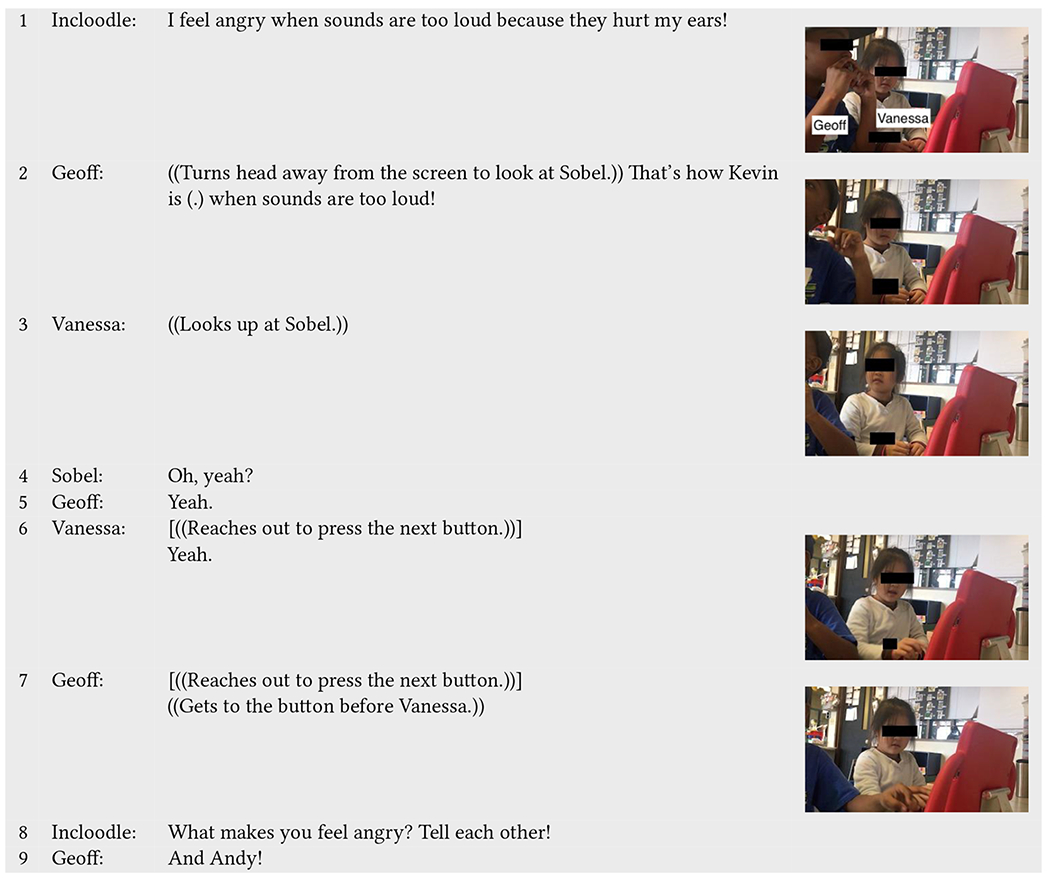

**Transcript 3.** Lisa (neurotypical) and Adam (neurodivergent) want to take a picture but with different shapes.

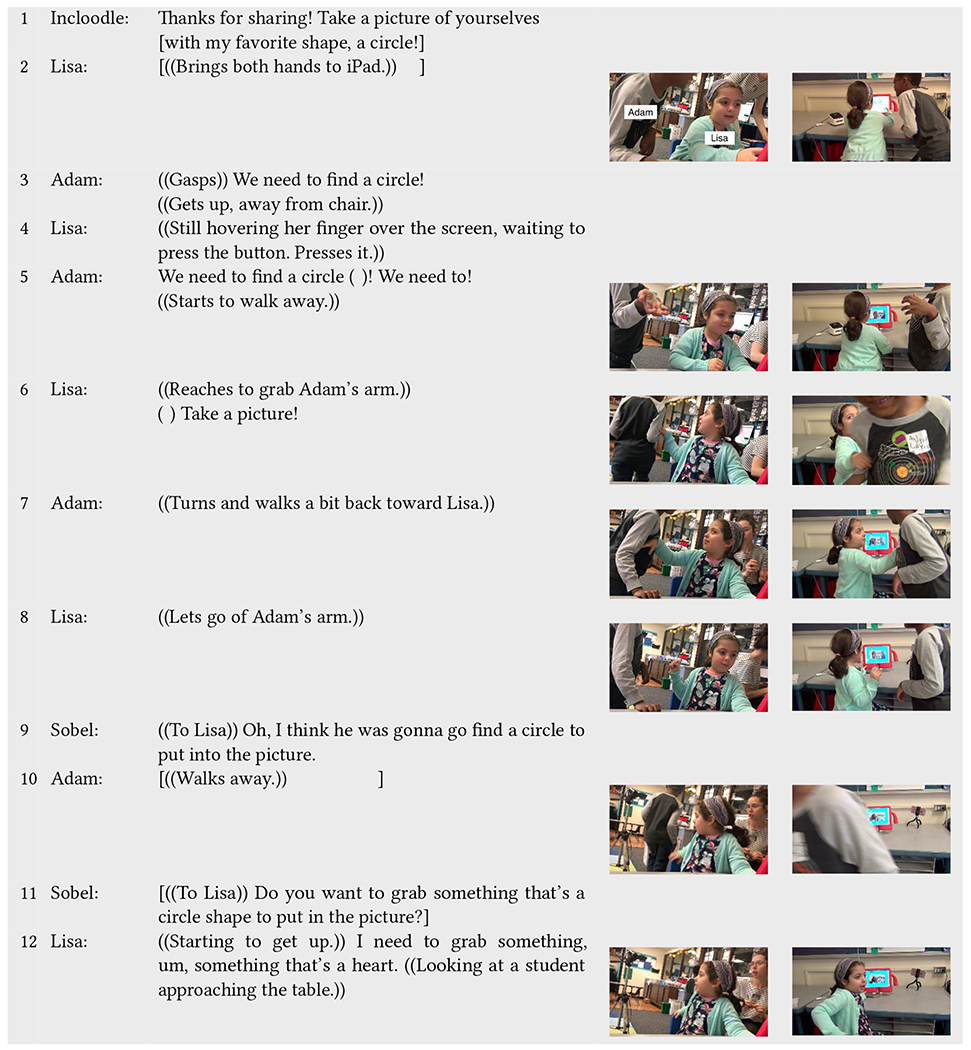

**Transcript 4.** Tristan (neurotypical) and Jeremy (neurodivergent) take a picture making grumpy faces.

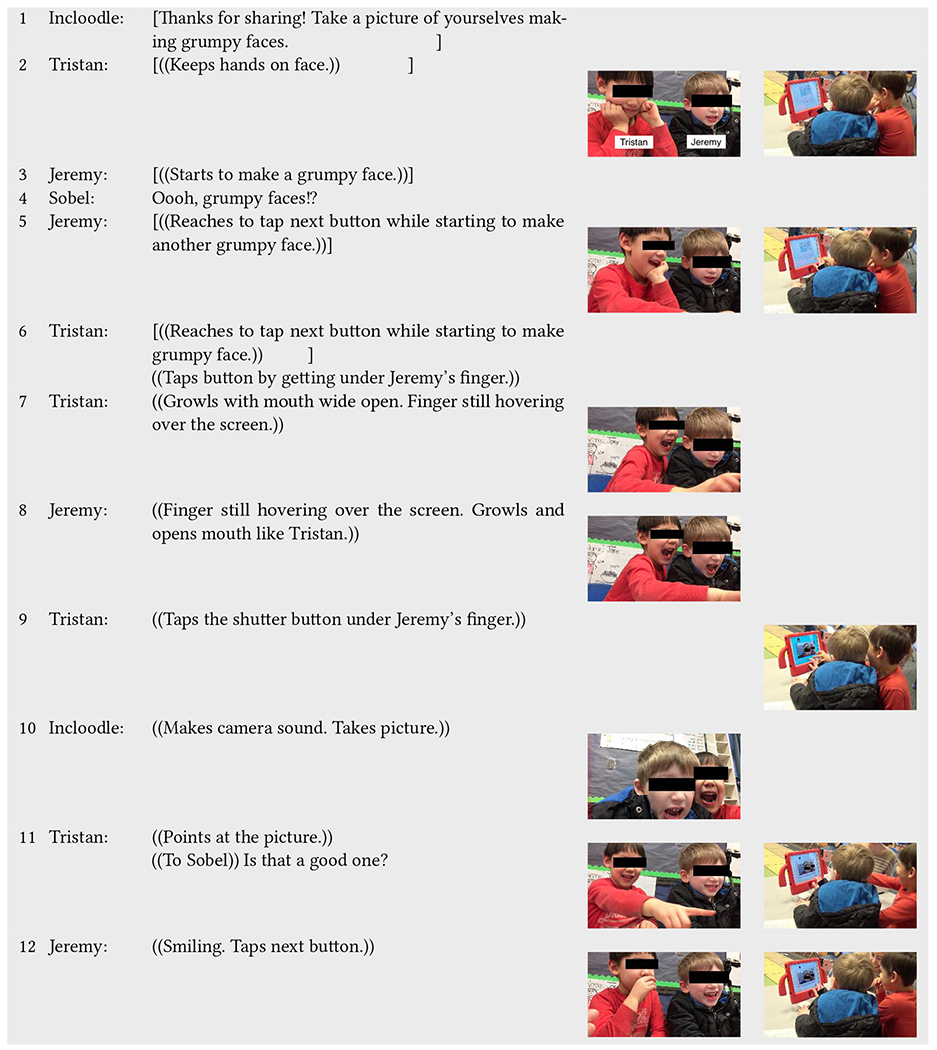

**Transcript 5.** Lisa (neurotypical) and Adam (neurodivergent) take a photo together showing different shapes.

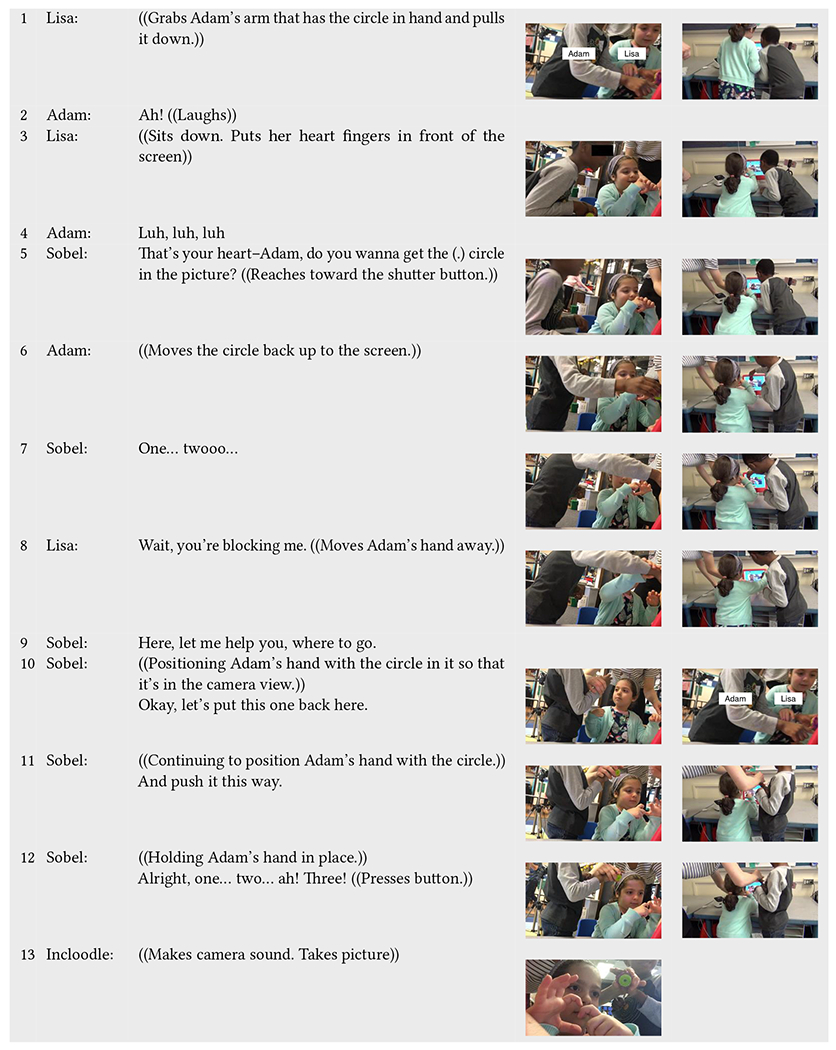

**Transcript 6.** Sam (neurotypical) repositions Incloodle camera to take a photo with Howie (neurodivergent).

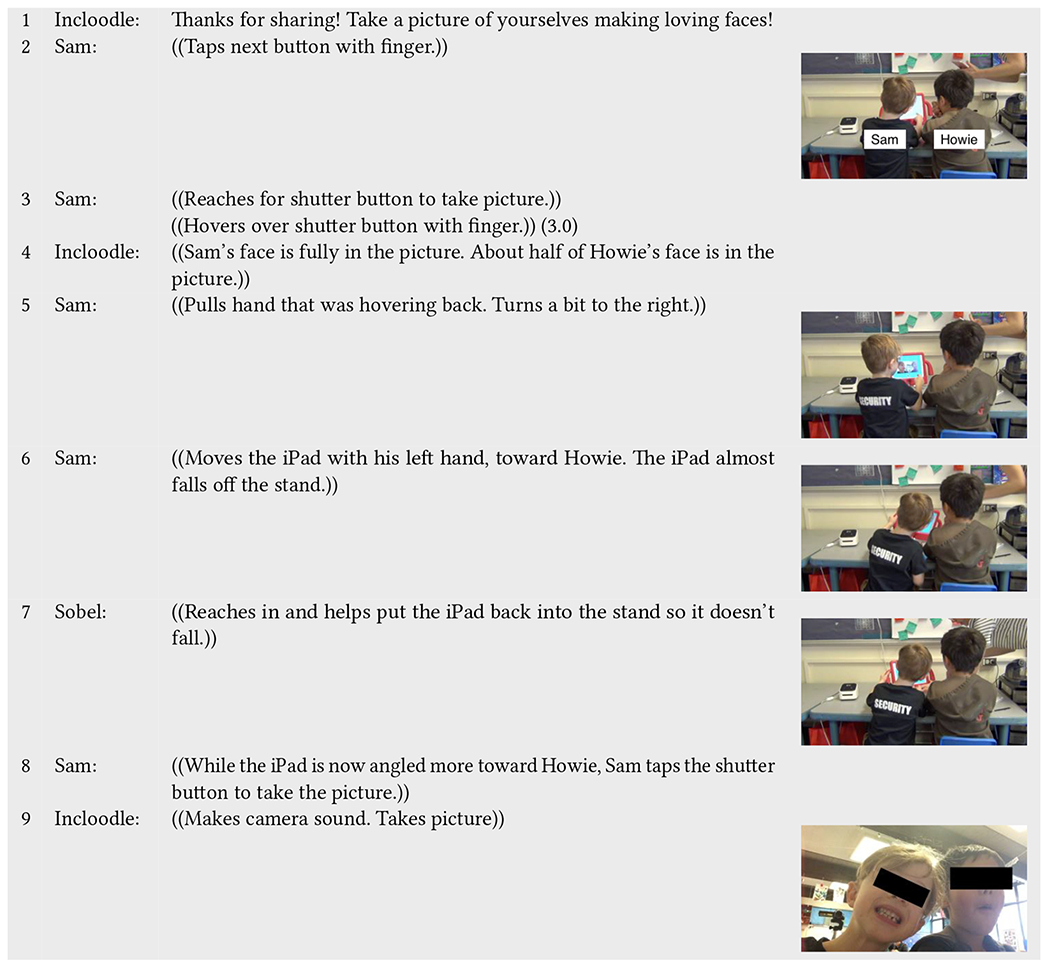

**Transcript 7.** Claire (neurotypical) and Gabe (non-speaking) take a photo with the help of adults.

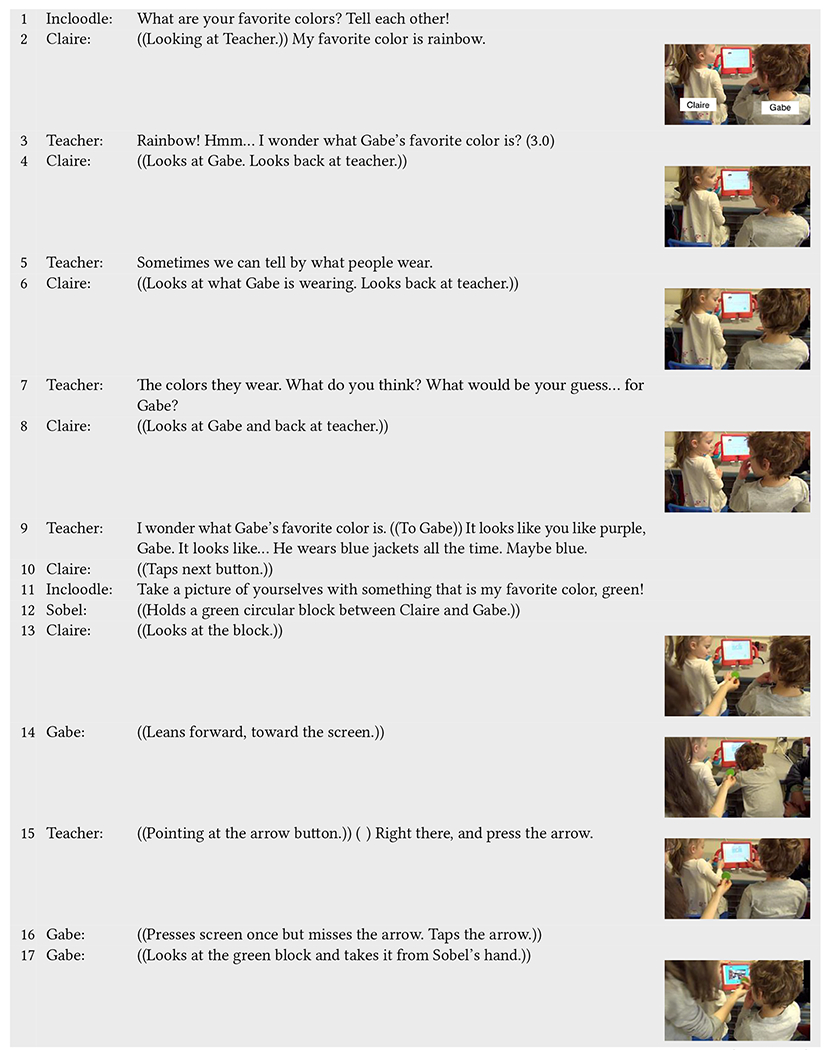
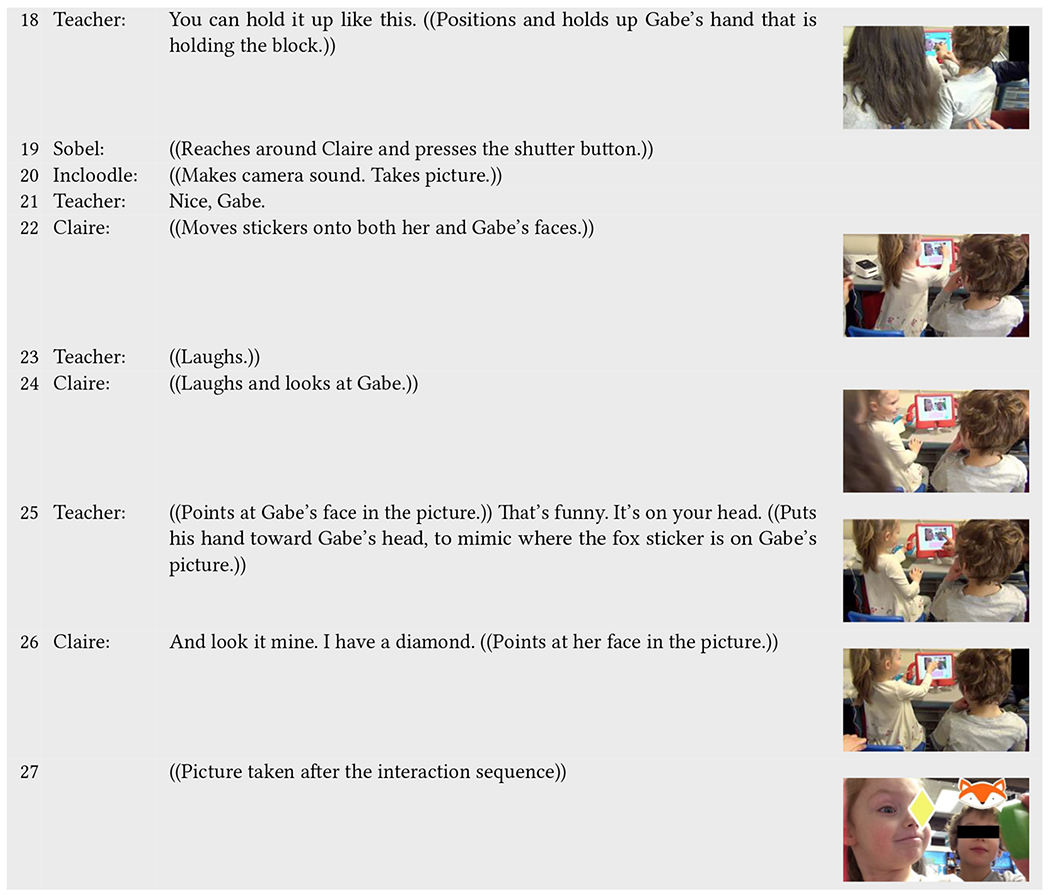

**Transcript 8.** Tristan (neurotypical) and Howie (neurodivergent) talk about their favorite numbers.

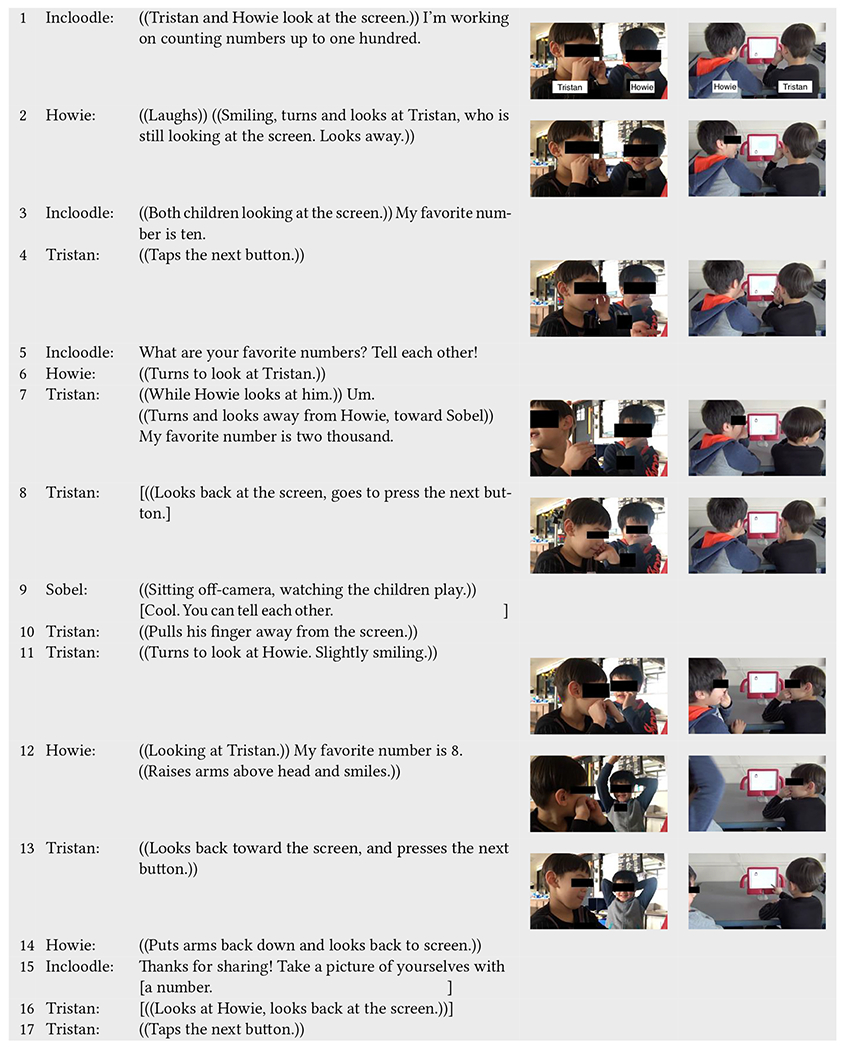
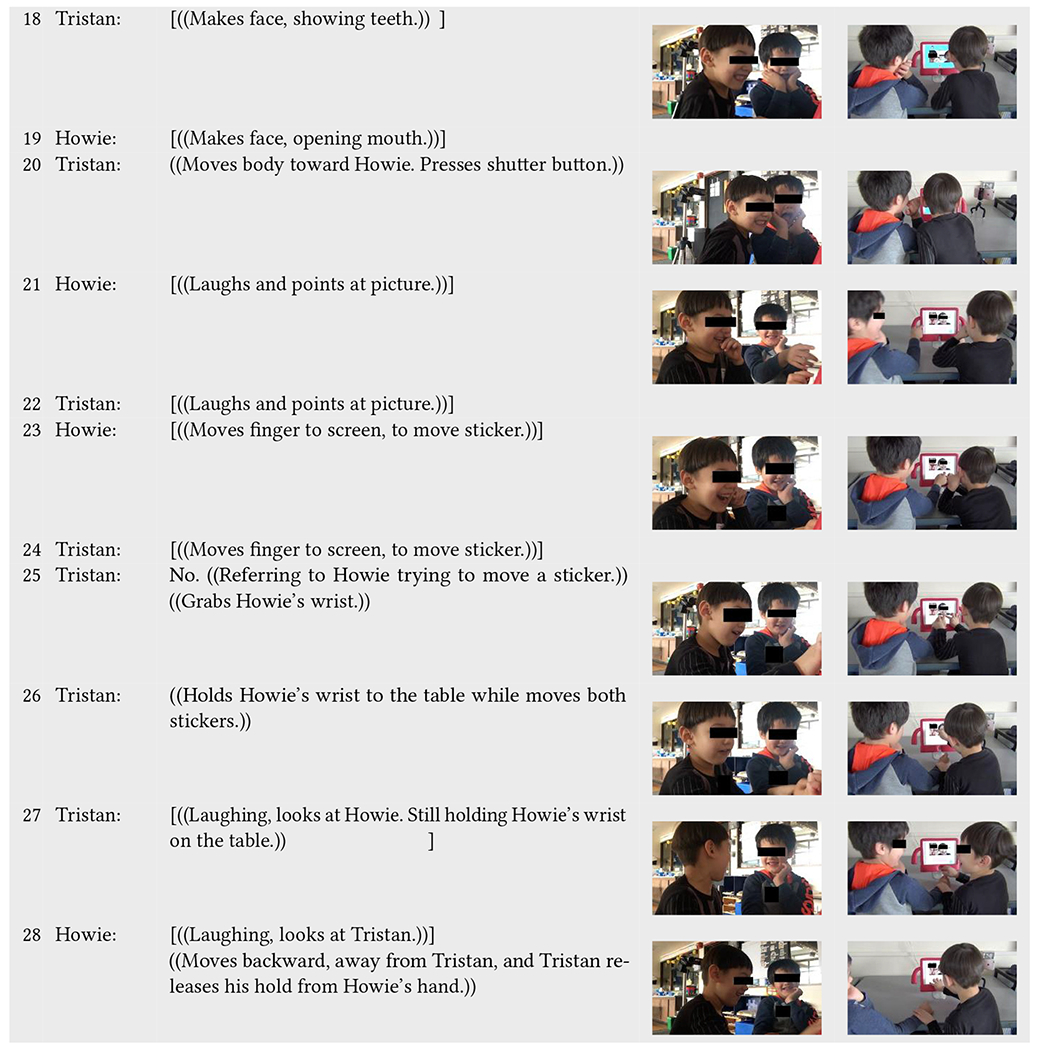

**Transcript 9.** Alex (neurodivergent) and Zoe (neurotypical) print the pictures they took using Incloodle.

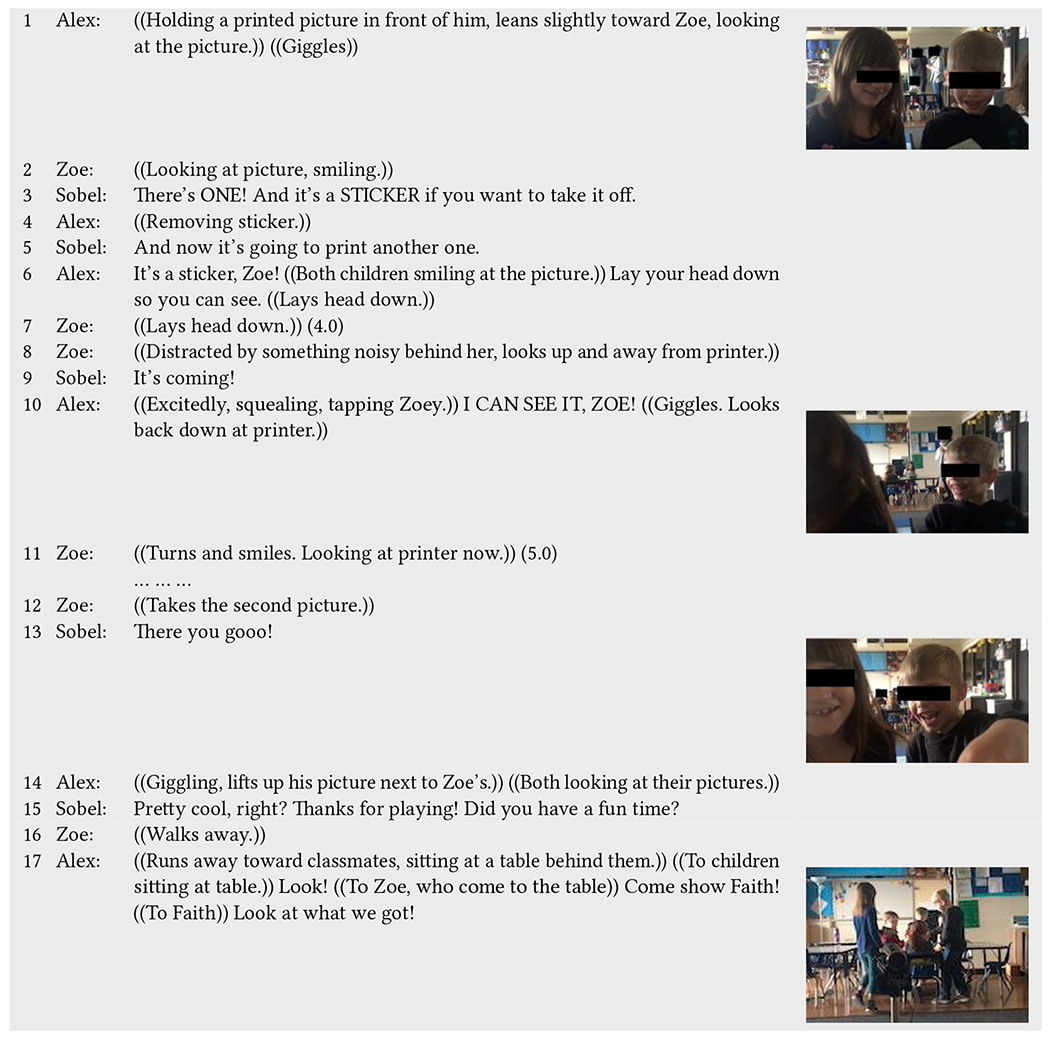

**Transcript 10.** Dylan (neurodivergent) and Geoff (neurotypical) discuss which photos to print.

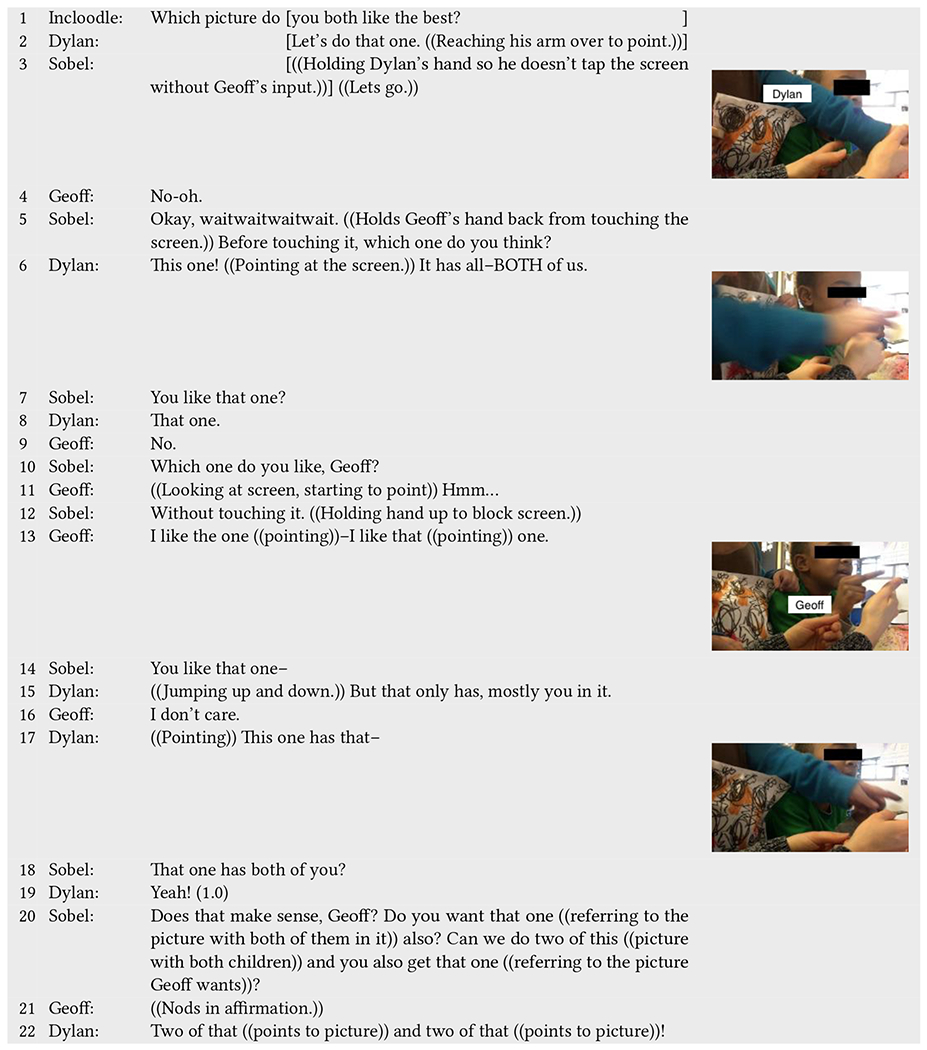

**Transcript 11.** Dylan (neurodivergent) and Geoff (neurotypical) print selected photos.

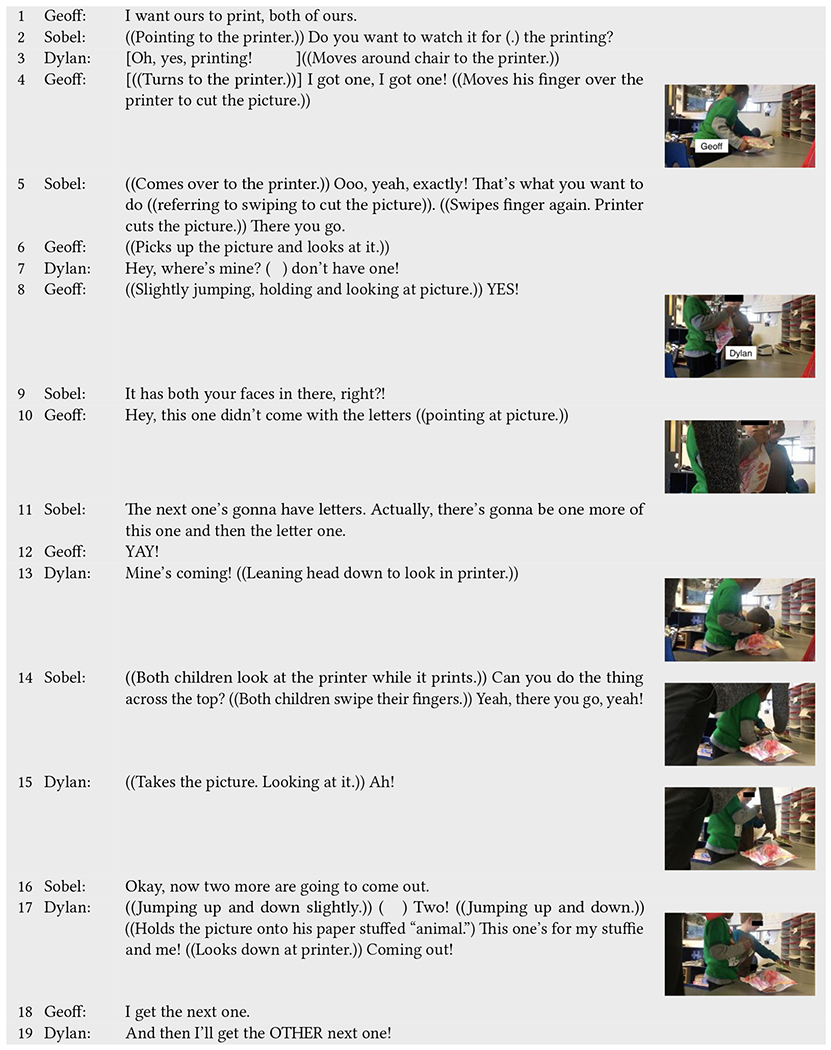

**Transcript 12.** Sobel repositions camera while Russell (neurodivergent) and Zoe (neurotypical) take a photo.

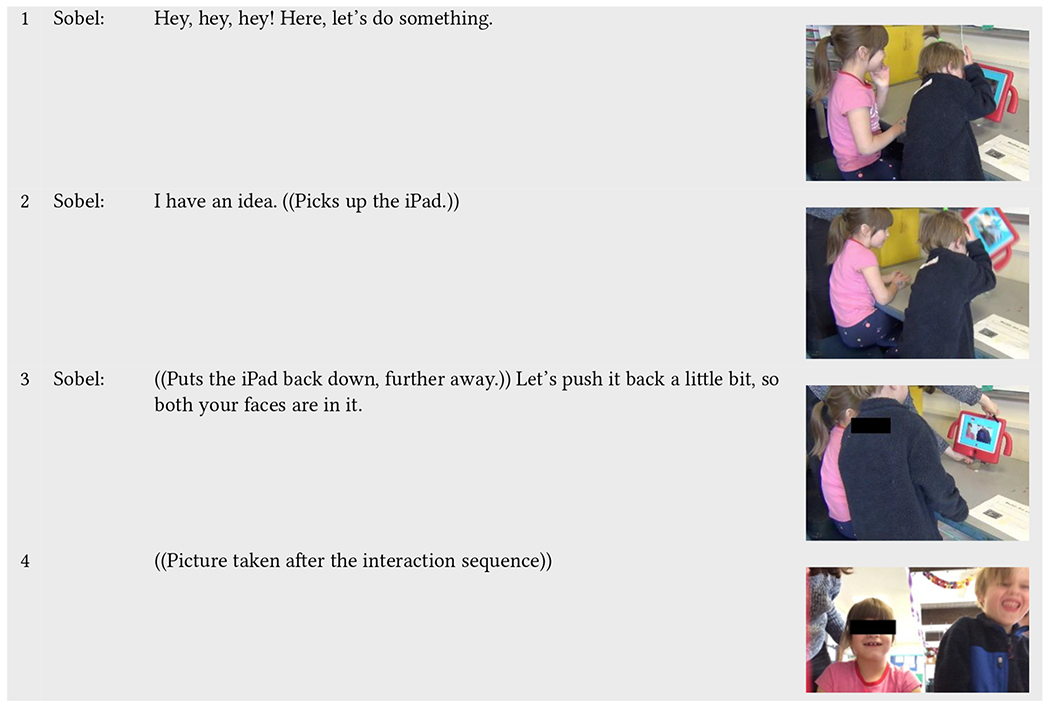

**Transcript 13.** Zoe (neurotypical) and David (neurodivergent) take a photo but David’s face is not in it.

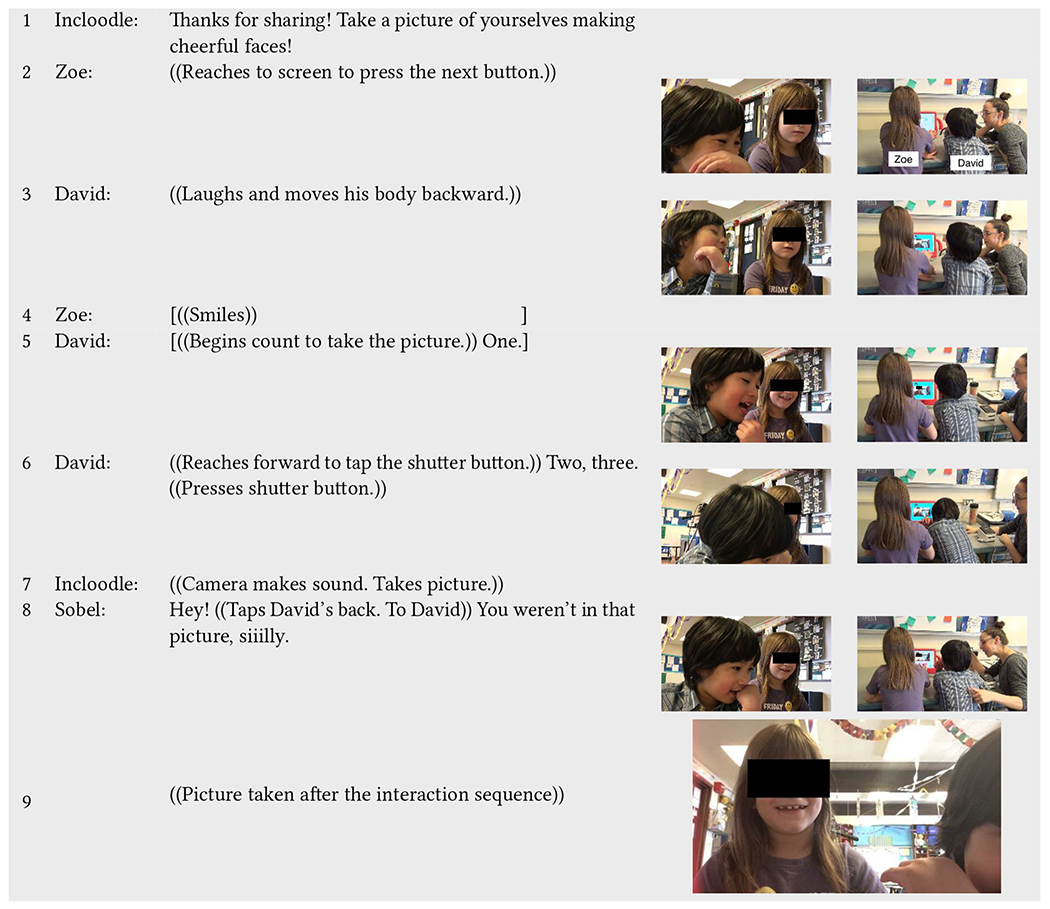

## CCS Concepts:

• Human-centered computing → Collaborative interaction; Empirical studies in accessibility;

## Footnotes

1 We use the term *neurodiverse* to describe heterogeneous groups that include children who have typical (i.e., *neurotypical*) and children who have atypical (i.e., *neurodivergent*) neurocognitive functioning.

2 Throughout this article, we use *Incloodle-Classroom* and *Incloodle* interchangeably to refer to the tablet app we developed and deployed in a kindergarten classroom. A previous version of the app is called *Incloodle 1.0* [79].

3 Individualized Education Program, also known as IEP, is a program developed to ensure that every child with a disability attending an elementary or secondary educational institution receives specialized instruction and related services under the Individuals with Disabilities Education Act.

4 Note that aligning with individual parent’s consent, we partially blur some children’s faces while not blurring others.

5 We’re Amazing 1, 2, 3! authored by Leslie Kimmelman and illustrated by Marybeth Nelson is a Sesame Street Big Golden Book about Julia, a new Sesame Street Muppet with autism.

6 It is important to note that Sobel consistently intervenes in this interaction sequence so neither child touches the screen. This is because, at this point of the design of Incloodle-Classroom, there was no way to go back and change the selected picture to print. Therefore, Sobel attempted to mediate children’s selection of pictures such that one child would not tap on a picture immediately without input from their play partner. If this happened, Sobel would have to exit out of Incloodle and let children print from the iOS photos app instead.

7
https://pbskids.org/games/feelings

8
https://www.joinkidmap.org/

## Appendix

**Table 1.** Incloodle-Classroom Content. Original Character Avatar Images Created by ©Liz Aragon. Final Images Edited and Produced by Lucas Colusso, Mackenna Lees, and Kiley Sobel

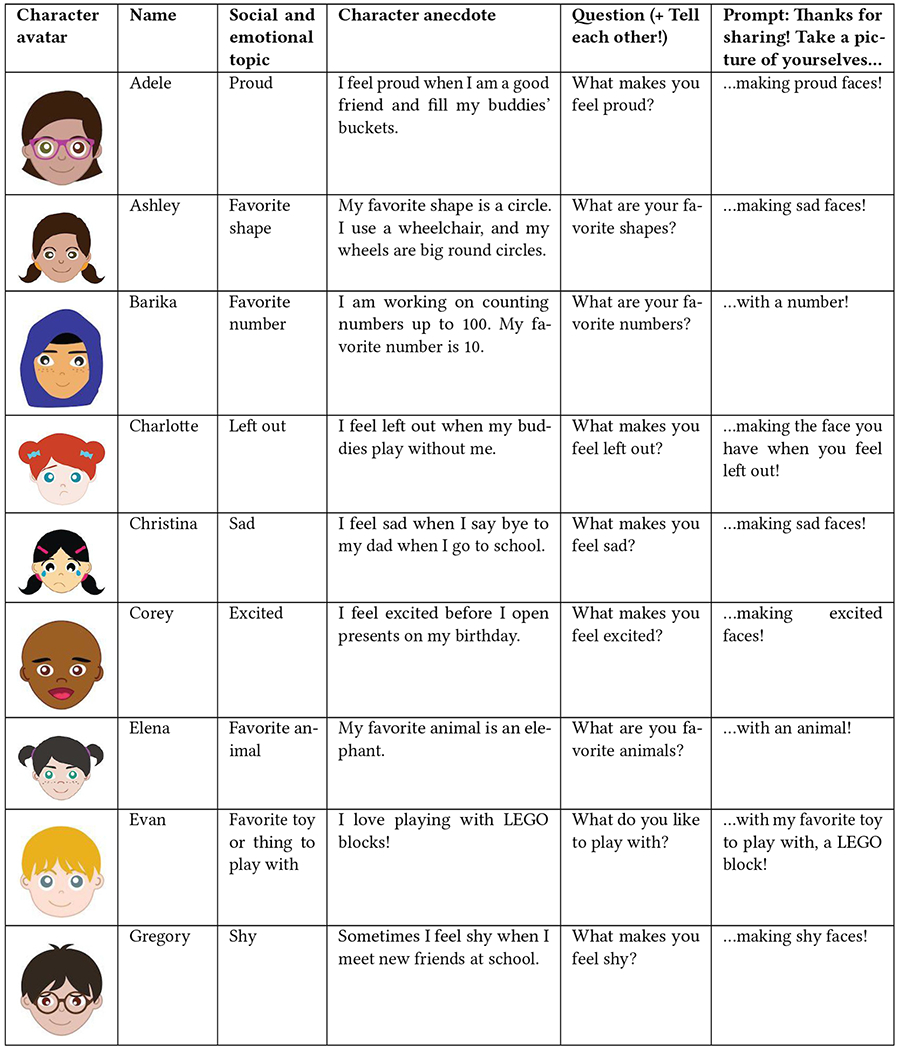

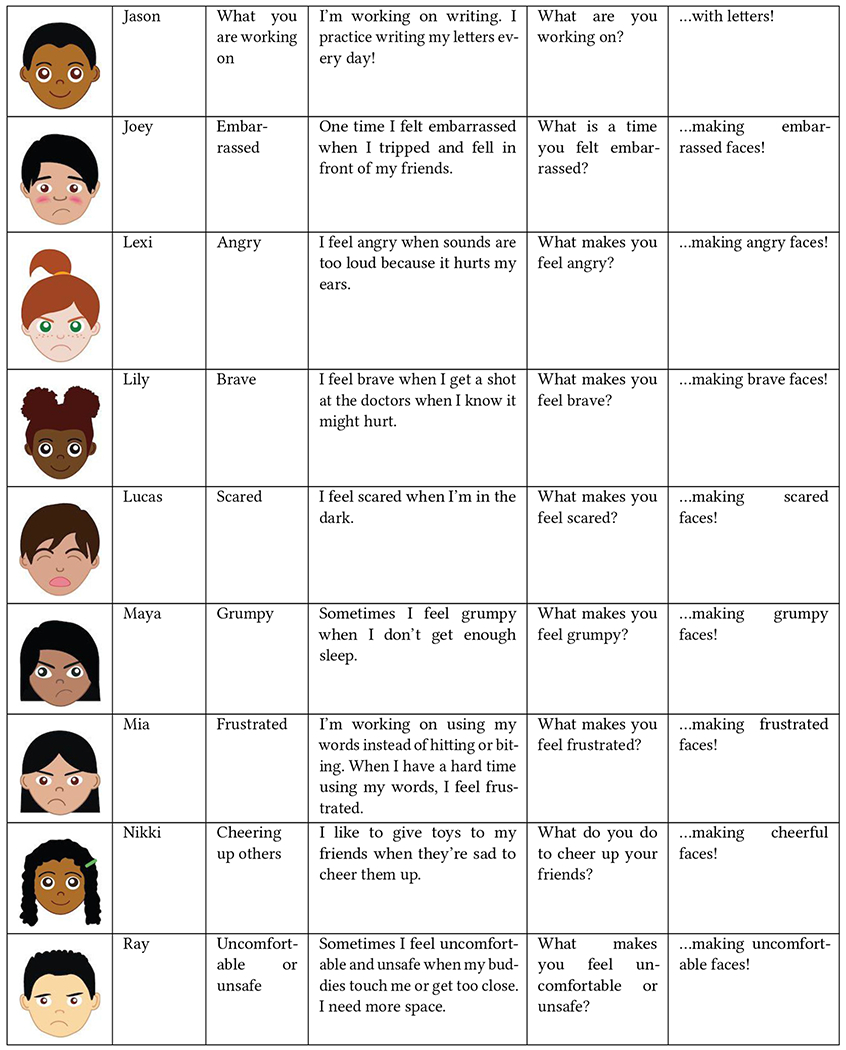

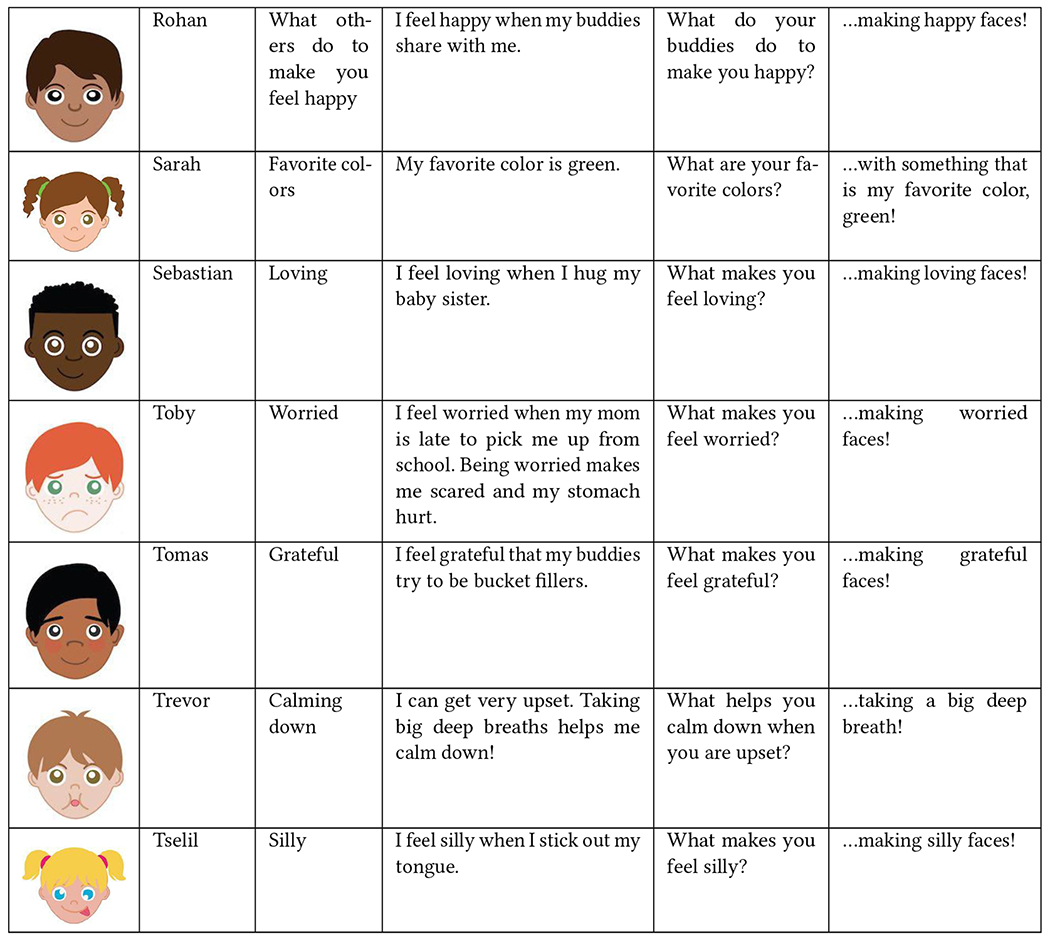

**Table 2.** Transcription Conventions

[	A *left bracket* indicates where an overlap begins.
]	A *right bracket* indicates where an overlap ends.
(.)	A *dot in parentheses* indicates a brief gap between utterances.
(( ))	*Double parentheses* contain the transcriber’s descriptions of non-verbal behavior.
()	*Empty parentheses* indicate the transcriber was unable to hear and transcribe what was being said.
(0.0)	*Numbers in parentheses* indicate time elapsed in seconds and tenths of a second.
–	A *dash* indicates a cutoff.
WORD	*Uppercase* letters or words indicate louder talk relative to the surrounding talk.
